# The effectiveness and cost-effectiveness of cash plus interventions to prevent acute malnutrition in Somalia: evidence from an adaptive cluster randomised control trial

**DOI:** 10.7189/jogh.16.04111

**Published:** 2026-05-08

**Authors:** Nadia Akseer, Kemish Kenneth Alier, Samantha Grounds, Sydney Garretson, Sagal Mohamud, Qundeel Khattak, Marina Tripaldi, Fabrizio Loddo, Said Aden Mohamoud, Adan Yusuf Mahdi, Mohamoud Ali Nur, Sadiq Mohamed Abdiqadir, Emily Mitchell, Andreas Kees, Mohamed Billow Mahat, Maimun Gure, Dahir Isaq Jibril, Dahir Gedi, Michael Ocircan P’Rajom, Meftuh Omer, Abdullahi Farah, Mohamed Abdirashid Osman, Abdiaziz Mohamed Adan, Farhan Mohamed Mohamoud, Abdullahi Muse Mohamoud, Abdifatah Ahmed Mohamed, Abdulkadir Ali Abdi, Adam Abdulkadir, Shelley Walton

**Affiliations:** 1 Department of International Health, Johns Hopkins University, Baltimore, Maryland, USA; 2 Save the Children, Save the Children International, London, UK; 3 Save the Children, Somalia Country Office, Mogadishu, Somalia; 4 Federal Government of Somalia, Ministry of Health and Human Services, Mogadishu, Somalia

## Abstract

**Background:**

Acute malnutrition affects millions of children aged <5 years, as well as pregnant and lactating women globally, especially in humanitarian settings. Although cash plus interventions – cash or food transfers combined with complementary components – are widely implemented, evidence remains limited on which combinations and durations are most effective at preventing acute malnutrition.

**Methods:**

We conducted a three-arm cluster-randomised trial within the ‘Save the Children’s Cash Plus for Nutrition’ programme in Somalia. Monthly support was provided as cash alone (arm 1), cash plus social and behaviour change communication (arm 2), or cash plus an additional cash top-up (arm 3). Further, we randomised 33 villages across arms, targeting approximately 1500 households. Primary outcomes included the prevalence and incidence of acute malnutrition in children aged 6–59 months and in mothers, assessed at baseline, midline (three months), and endline (six months). We also conducted market monitoring, qualitative data collection, and analysis.

**Results:**

Child acute malnutrition prevalence was approximately 15.0% in each arm at baseline. After three months, prevalence declined by 2.0 percentage points in arm 1 to 13.0% (95% confidence interval (CI) = 10.3, 16.1), representing a relative reduction of 13.3%. In arm 2, prevalence declined by 5.9 percentage points to 9.1% (95% CI = 6.8, 11.8), a relative reduction of 39.3%. In contrast, prevalence in arm 3 remained essentially unchanged, increasing to 15.1% (95% CI = 12.1, 18.6). By endline, there was little change from midline in all arms. Maternal malnutrition improved most in arm 2, but differences were not statistically significant. All arms showed improvements in dietary diversity and food security, but only arm 2 achieved sustained nutrition gains. Household livelihood conditions appeared to improve overall, though monthly expenditures nearly doubled. Arm 2 was the most effective and cost-effective.

**Conclusions:**

Adding social and behaviour change communication to cash transfers significantly improved child nutrition compared to cash alone, highlighting that integrated approaches can enhance nutritional outcomes and at minimal additional cost in humanitarian settings.

**Registration:**

ClinicalTrials.gov: NCT06642012.

Somalia, an impoverished country that has experienced years of conflict, violence and environmental disasters, continues to suffer from complex emergencies that result in large-scale population displacement, disease and food insecurity [1]. Extreme drought between 2020 and 2022, and severe flooding in 2023, have reduced agricultural production and damaged critical healthcare and water, sanitation, and hygiene infrastructure [1]. Despite an ongoing humanitarian presence, the Integrated Food Security Phase Classification (IPC) estimated that four million people (21% of Somalia’s population) were living in crisis or emergency states of food insecurity from January–March 2024 [2].

Children aged <5 years are especially vulnerable to food insecurity due to increased nutritional needs during this critical period of child development, and food-insecure children have a heightened risk of acute malnutrition (wasting), morbidity, and mortality [3]. Estimates of wasting prevalence among children aged <5 years in Somalia in 2009 and 2019 ranged from 11.0–14.3% – more than double the global wasting rate of 6.8% [4,5]. The IPC projects that 1.7 million children aged <5 years (approximately 45% of all children aged <5 years) in Somalia will suffer from acute malnutrition from January to December 2024 [2].

In 2023, the United States Agency for International Development (USAID) provided USD 761 million to Bureau for Humanitarian Assistance (BHA) relief partners in Somalia for food and cash assistance, medical services and treatment for wasting, and water, sanitation, and hygiene support [6]. Multipurpose cash programming has been established as the preferred modality for flexibly meeting the basic needs of Somali households [7]. In 2023, an estimated 8.3 million people in Somalia required this type of humanitarian assistance [1,8].

Cash and vouchers assistance (CVA), also known as cash transfers, has been increasingly used to address household food security and nutrition in humanitarian settings such as Yemen, Niger, and Somalia [3,9–19]. However, evidence on the effectiveness and cost-effectiveness of these interventions for preventing wasting, especially when implemented in such complex and fragile settings, requires further study [9,11,12]. CVA has proven cost-effective for improving food security, especially through mobile and direct delivery payment mechanisms that reduce logistical costs, thereby making cash a viable option even in challenging humanitarian contexts [20,21]. However, existing evidence on cash transfer interventions has primarily focused on food security, with limited documentation of their impact and effectiveness on nutrition outcomes and the prevention of acute malnutrition [3,17,18,22].

When combined with education or behaviour change support, cash transfers can further enhance food security and dietary intake outcomes by enabling recipients to make informed choices about their food needs [23]. Although the role and importance of social and behaviour change communication (SBCC) in complementing cash voucher assistance have increasingly been recognised, there is a need for further evidence on the effectiveness and cost-effectiveness of cash plus SBCC approaches for improving nutrition outcomes in humanitarian settings [19,24–27].

In Somalia, a few non-randomised trials have measured the impact of CVA on nutrition outcomes for children aged <5 years [28,29], but did not include a cash plus SBCC intervention arm, explore the impact of different cash amounts, perform cost-effectiveness analyses, or use weight-for-height Z-score (WHZ) as the gold standard for assessing child wasting, which may underestimate true wasting burden [30–33]. More importantly, the trials lacked the rigour afforded by a randomised controlled trial design [3,13]. While a quasi-experimental trial in Somalia measured the impact of a cash plus nutrition counselling intervention on child nutrition outcomes, the study did not conduct any cost analyses and lacked a cash-only intervention arm for comparison [14]. Moreover, market accessibility, functionality, and diversity are essential components of a successful cash-for-nutrition programme. Without functioning and diverse markets, cash transfers may not improve nutrition, as beneficiaries may lack access to nutritious food options or face inflated prices due to market inefficiencies. Yet, research on this area is limited.

Given these knowledge gaps, the need for additional research using randomised controlled trial design and mixed methods studies to assess the impact and cost-effectiveness of various cash and ‘cash plus SBCC’ interventions in humanitarian settings has been emphasised [3,12,13,15–19,23,34,35].

We aimed to address the evidence gap regarding the effectiveness and cost-effectiveness of cash plus approaches by conducting a three-arm cluster-randomised trial embedded within a large Cash Plus for Nutrition programme implemented by Save the Children in Somalia. The programme is implemented in camps supporting internally displaced persons (IDP) in two high-burden regions of Somalia (Bay and Hiran). Specifically, our aims were to estimate and compare acute malnutrition incidence and prevalence of children aged 6–59 months old and their mothers receiving each month either cash (arm 1 – control), cash plus SBCC (arm 2), or cash plus top-up cash (arm 3); after three months and six months of cash assistance. Further, we aimed to calculate the costs and cost-effectiveness of the different intervention arms and to understand determinants that may influence the effectiveness of the programme (the perspectives and experiences of mothers and fathers of children aged <5 years who are beneficiaries of the cash programme, and the functionality of markets and food prices).

The ongoing food insecurity crisis in Somalia necessitates continued humanitarian support, and investigating cost-effective cash assistance interventions is of the utmost importance to implementers and programme partners. This study builds upon the evidence base through an adaptive, randomised controlled trial design, the incorporation of cost-effectiveness analyses, and rigorous anthropometric measurements, including WHZ. These findings will promote evidence-based decision-making and inform streamlined programmatic approaches for addressing malnutrition in Somalia and other humanitarian contexts.

## METHODS

### Study design

We used a mixed-methods design that included a prospective, adaptive, cluster-randomised controlled trial, qualitative data collection, market monitoring, and costing analyses, including cost-efficiency and cost-effectiveness analyses [36]. An adaptive design was adopted for the trial to enable flexibility in adjusting the design and other parameters as needed in the complex, unpredictable humanitarian setting [37–39]. The United Nations International Children’s Emergency Fund (UNICEF) Conceptual Framework on Maternal and Child Nutrition categorises the determinants of malnutrition into three groups – immediate, underlying, and basic/enabling causes [27,34,40,41]. This evidence-based conceptual framework informed the trial design and both quantitative and qualitative data collection and analysis.

### Study context

In the Horn of Africa, Somalia experiences an arid/semi-arid climate defined by four alternating rainy and dry seasons [42,43]. This study took place in Baidoa (the capital of the Bay region) and in three districts of the Hiran region in central Somalia – Beledweyne (regional capital), Mataban, and Mahas. Both Bay and Hiran comprise urban, rural, nomadic, and internally displaced populations, with the majority of the population in both settings being rural or nomadic [44,45]. Around a quarter of Somalia’s IDP are hosted in Baidoa, with IDP populations experiencing the highest burden of acute malnutrition [46,47].

Conflict, insecurity, droughts, and floods have affected Bay and Hiran for years, undermining livelihoods and food security, contributing to displacement, and resulting in high rates of acute malnutrition in these regions [47–51] (Figure S1 in the **Online Supplementary Document**).

### BHA Cash Plus for Nutrition programme

Save the Children implemented the USAID/BHA-funded Cash Plus for Nutrition programme in the Bay and Hiran regions of Somalia, which included unconditional cash transfers (UCTs) and social behaviour change communication interventions to families largely based in IDP settings. The cash-for-nutrition implementation model followed Save the Children’s common approach, Resourcing Families for Better Nutrition, in which unconditional cash is combined with a comprehensive set of context-specific, tailored social and behaviour change interventions for the target group [27]. The SBCC package designed for this research is summarised in Table S1 and Text S1 in the **Online Supplementary Document**. Further programme details are available in the methods paper [36].

### Trial design

In consultation with key stakeholders and in light of evidence gaps, we implemented the following three arms, each using UCTs as the base intervention. Arm 1 – UCT to targeted families (one mobile transfer per month for six consecutive months). Households in Bay received USD 90 per month, and households in Hiran received USD 70 per month. Arm 2 – UCT with SBCC (cash was the same amount as arm 1, coupled with the SBCC package). The SBCC package was context-specific and tailored to pregnant and breastfeeding women and children aged <5 years. This package included interpersonal communication, such as one-to-one counselling for mothers of children aged <5 years, and bimonthly group sessions of approximately 45–60 minutes through mother-to-mother support groups, covering topics related to nutrition, health, and community awareness-raising campaigns. Participants were enrolled in mother-to-mother support groups over two three-month cycles, totalling 12 sessions. Additionally, the implementing partner organised cooking demonstrations for the support groups, highlighting nutritious foods available in the community (Table S1 and Text S1 in the **Online Supplementary Document**) [36]. Arm 3 – UCT with additional monthly nutrition-cash top-up (one per month for six months). Households in Bay received USD 90 plus an additional USD 35 top-up (USD 125 total) per month, and households in Hiran received USD 70 plus an additional USD 35 top-up (USD 105 total) per month.

The base cash transfer amounts were established by the National Cash Working Group, using the food minimum expenditure basket. Cash amounts were calculated to meet 80% of a person’s monthly food energy needs (calories) and were based on the average number of household members [32,49,50]. The arm 3 top-up cash transfer amount was aligned to the cash top-up provided by the World Food Program for nutrition. It was calculated by the World Food Program using the fill-the-nutrient-gap methodology to cover the additional nutritional needs of pregnant and lactating women and children aged <5 years while considering the available nutritious food in the project-targeted locations and households’ capacities to meet their food needs.

### Study population, randomisation and sample size

We randomised clusters (distinct villages) to one of three arms and then selected households within those arms as follows: an initial 44 clusters were identified for randomisation, which was then reduced to 33 based on accessibility and feasibility for research. The clusters were randomised to one of three arms using a random number generator. Households with children aged <5 years were identified from the BHA programme roster and were contacted to enrol in the study. Additional details available in the methods paper [36].

**Figure 1:**
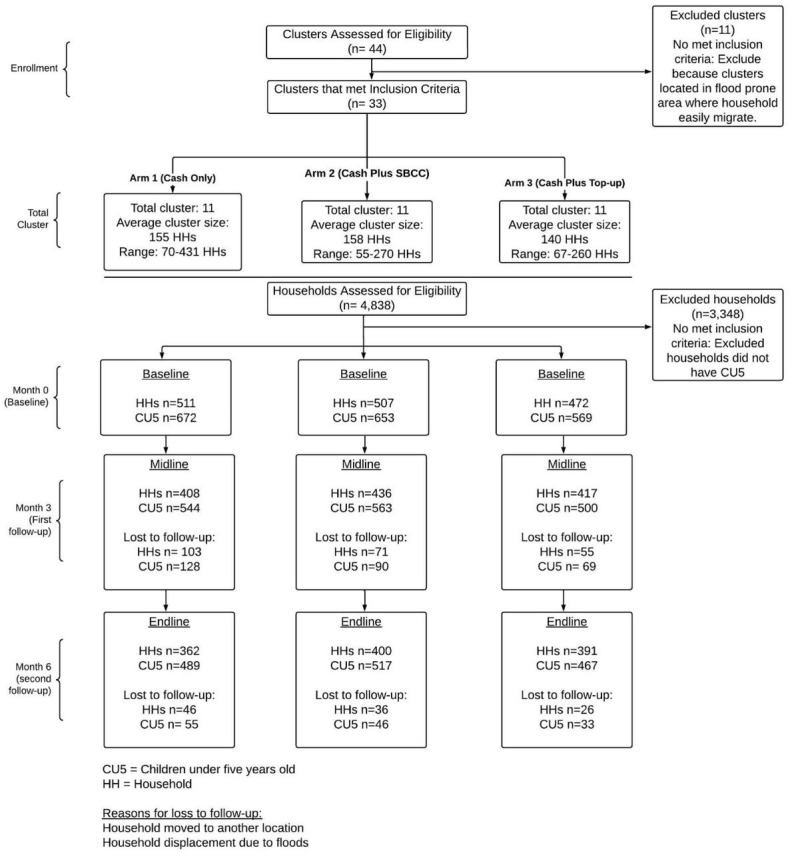
CONSORT flowchart for study enrolment and follow-up.

We estimated the number of households needed for the trial using child wasting as the primary outcome, considering feasibility/logistics in study settings, and a 7% minimum detectable difference in post-intervention prevalence of wasting. We assumed: 20% baseline wasting, intra-class correlation coefficient from earlier studies (0.02) [29,52], average cluster size (150 households), number of clusters (33 total, 11 per arm), 5% significance and 80% power, and children aged <5 years per household (1.3 children). These parameters yielded 410 households per intervention group, or 533 children. Accounting for 15% attrition, the final required sample size was 471 households or 613 children per arm. We evaluated a total of 4838 households for eligibility against the trial’s inclusion and exclusion criteria. We excluded 3348 households because they did not have children aged <5 years or did not meet one or more exclusion criteria (Table S2 in the **Online Supplementary Document**). The final sample included 1490 households with 1894 children, approximately 569–672 children across the arms (**Figure 1**).

### Outcomes

The primary outcomes of interest were child wasting, defined by mid-upper arm circumference (MUAC), WHZ, bilateral pitting oedema, and maternal wasting, defined by MUAC. WHZ was our primary analytical outcome, and we calculated it according to the 2006 World Health Organization (WHO) Child Growth Standards [53–55]. We coded values outside the WHO plausible ranges as missing: WHZ<–5 or WHZ>5, weight-for-age Z-score (WAZ)<–6 or WAZ>5, and height-for-age Z-score (HAZ)<–6 or HAZ>6. Child wasting was categorised as a binary outcome (wasted/not wasted) and as a categorical outcome, with wasting stratified into moderate (MAM) or severe wasting. Maternal wasting was categorised as overweight, normal, or moderate acute malnutrition [56]. A secondary outcome of interest was child stunting, defined by HAZ. For children who were older than 59 months at midline and endline follow-up, anthropometric measurements were standardised to 59 months [52,57,58] (Table S3 in the **Online Supplementary Document**).

### Data collection and analysis

The trial was implemented from June to November 2023. Baseline quantitative data collection occurred at the end of May 2023, midline at mid-September 2023, and endline at mid-December 2023. We collected qualitative data in January 2024. Local enumerators were recruited and underwent comprehensive training prior to baseline data collection. Standard procedures and equipment were used to collect data (Text S2 in the **Online Supplementary Document**).

Along with outcome data, the quantitative survey instrument implemented at each time point collected information on immediate, underlying, and enabling causes of malnutrition [59,60] (Table S4 in the **Online Supplementary Document**).

In this study, we employed a mixed-methods approach encompassing quantitative, qualitative, economic, and market analyses (Tables S5–7, Texts S3–5 in the **Online Supplementary Document**) [36]. We rigorously cleaned quantitative data and validated it in near real time, with the main analyses conducted using an intention-to-treat (ITT) approach and complemented by per-protocol and incidence analyses to assess the robustness of the findings. Multilevel mixed-effects models were used to estimate treatment effects, with support from a difference-in-differences logistic regression framework and sensitivity analyses addressing missing data, time specifications, and loss to follow-up. We calculated adjusted predictions and marginal effects, and all models adhered to CONSORT guidelines (Text S3 in the **Online Supplementary Document**). We collected qualitative data through focus group discussions with mothers and fathers to contextualise quantitative findings, using a sequential explanatory design (Text S3 and S4 in the **Online Supplementary Document**). Cost-efficiency and cost-effectiveness analyses triangulated findings from financial records, modelling, and staff consultations, applying a top-down, activity-based costing approach [61,62] that included societal costs (Table S5 and Texts S3–5 in the **Online Supplementary Document**). We conducted market monitoring at three time points, using vendor surveys in both regions, to track the availability and prices of key food commodities, enabling analysis of how contextual shifts may have influenced household behaviour and the impacts of interventions (Tables S6 and S7 and Text S3 in the **Online Supplementary Document**).

## RESULTS

We present the results by first describing the sample, then the key maternal and child outcomes (effectiveness and cost-effectiveness), and finally exploring potential factors that may influence the outcomes, including beneficiaries’ perspectives and market functionality.

### Baseline descriptive statistics

The sample comprised one-third from Bay and two-thirds from Hiran, with a similar distribution between IDP settings (one-third) and host communities (two-thirds). Arm 3 included slightly more participants from Bay and a higher proportion of IDPs (**Table 1**).

**Table 1:** Baseline characteristics of households, mothers, and children*

Characteristics	Trial arm			*P*-value
	**1 (cash only) (n = 672)†**	**2 (cash plus SBCC) (n = 653)†**	**3 (cash plus cash top-up) (n = 569)†**	
Region				
*Bay*	252 (37.5)	250 (38.3)	295 (51.8)	<0.001
*Hiran*	420 (62.5)	403 (61.7)	274 (48.2)	
Displacement status				
*Host*	416 (61.9)	403 (61.7)	271 (47.7)	<0.001
*IDP*	256 (38.1)	250 (38.3)	297 (52.3)	
Child sex				
*Male*	357 (53.1)	318 (48.7)	288 (50.6)	0.271
*Female*	315 (46.9)	335 (51.3)	281 (49.4)	
Child age in months, x̄ (SD)	34.4 (14.6)	34.7 (14.3)	33.7 (13.9)	0.507
Age in years				
<2	162 (24.1)	150 (23.0)	134 (23.6)	0.888
>2	510 (75.9)	503 (77.0)	435 (76.4)	
Child MUAC in cm, x̄ (SD)	14.3 (1.0)	14.5 (1.0)	14.3 (1.2)	0.069
WHZ-score	–0.9 (1.1)	–0.9 (1.0)	–0.9 (1.1)	0.963
WAZ-score	–1.2 (0.9)	–1.1 (0.9)	–1.3 (0.9)	0.047
HAZ-score	–1.0 (1.3)	–1.0 (1.3)	–1.2 (1.4)	0.007
Wasting				
*WHZ≥–2 SD*	567 (85.3)	554 (85.0)	480 (84.5)	0.933
*WHZ<–2 SD*	98 (14.7)	98 (15.0)	88 (15.5)	
Wasting (MUAC) in cm				
*≥11.5*	670 (99.7)	652 (100.0)	567 (99.6)	0.342
*<11.5*	2 (0.3)	0 (0.0)	2 (0.4)	
Underweight				
*WAZ≥–2 SD*	554 (82.7)	556 (85.1)	455 (80.0)	0.058
*WAZ<–2 SD*	116 (17.3)	97 (14.9)	114 (20.0)	
Stunting				
*HAZ≥–2 SD*	537 (80.4)	534 (82.0)	410 (72.3)	<0.001
*HAZ<–2 SD*	131 (19.6)	117 (18.0)	157 (27.7)	
Child ever breastfed (youngest child only 6–23 mo)‡				
*No*	18 (10.3)	27 (16.9)	18 (12.6)	0.198
*Yes*	157 (89.7)	133 (83.12)	125 (87.4)	
Received any childhood vaccination‡				
*No*	86 (49.1)	82 (51.6)	77 (53.8)	0.704
*Yes*	89 (50.9%)	77 (48.4%)	66 (46.2%)	
Had a childhood illness two weeks prior‡				
*No*	103 (58.9)	109 (68.6)	106 (74.1)	0.013
*Yes*	72 (41.1)	50 (31.4)	37 (25.9)	
Child minimum dietary diversity§				
*Do not meet*	149 (94.9)	117 (88.0)	112 (89.6)	0.093
*Meet*	8 (5.1)	16 (12.0)	13 (10.4)	
Animal-sourced protein (meat, eggs, milk)				
*No*	82 (46.9)	66 (41.3)	62 (43.4)	0.579
*At least one*	93 (53.1)	94 (58.7)	81 (56.6)	
Mother age in years, x̄ (SD)	32.2 (9.0)	34 (10.8)	32.9 (9.9)	0.014
Age in years				
*18–34*	306 (59.9)	281 (55.5)	278 (59.2)	0.325
*>35*	205 (40.1)	225 (44.5)	192 (40.8)	
MUAC in cm, x̄ (SD)	26.7 (4.0)	27.0 (3.8)	26.1 (3.6)	0.002
Maternal wasting (MUAC) in cm				
*<23*	67 (13.9)	55 (11.5)	69 (15.5)	0.193
*≥23*	415 (86.1)	424 (88.5)	375 (84.5)	
Pregnancy status				
*No*	420 (82.2)	400 (79.1)	370 (78.4)	0.277
*Yes*	91 (17.8)	106 (20.9)	102 (21.6)	
Education level				
*No formal education*	420 (82.2)	418 (82.6)	410 (86.9)	0.215
*Madrasa*	51 (10.0)	51 (10.1)	40 (8.5)	
*Primary and secondary*	40 (7.8)	37 (7.3)	22 (4.7)	
Mother is a head of household				
*No*	360 (70.5)	354 (70.0)	339 (71.8)	0.804
*Yes*	151 (29.5)	152 (30.0)	133 (28.2)	
Decision-making on income				
*Jointly*	215 (42.5)	205 (41.0)	199 (42.4)	0.95
*Mother*	111 (21.9)	117 (23.4)	100 (21.3)	
*Father*	180 (35.6)	178 (35.6)	170 (36.3)	
Decision-making on healthcare				
*Jointly*	248 (48.9)	266 (53.1)	254 (54.2)	0.188
*Mother*	100 (19.7)	109 (21.8)	90 (19.2)	
*Father*	159 (31.4)	126 (25.2)	125 (26.7)	
Number of children aged <5 y in the household				
*1*	351 (68.7)	367 (72.4)	373 (79.0)	0.001
*≥2*	160 (31.3)	140 (27.6)	99 (21.0)	
Household hunger scale				
*Little to no hunger (score ≤1)*	283 (55.4)	307 (60.7)	307 (65.0)	0.001
*Moderate hunger (score 2–3)*	219 (42.9)	197 (38.9)	165 (35.0)	
*Severe hunger (score 4–6)*	9 (1.7)	2 (0.4)	0 (0.0)	
Food consumption score (adjusted for high oils and sugar consumption)				
*Poor (score ≤28)*	262 (51.3)	231 (45.6)	189 (40.0)	0.007
*Borderline (score 28.5–42)*	124 (24.3)	131 (25.9)	125 (26.5)	
*Acceptable (score ≥43)*	125 (24.4)	144 (28.5)	158 (33.5)	
Reduced coping strategies index score (0 – best, 56 – worse), x̄ (SD)¶	14.5 (9.0)	15.3 (8.7)	14.9 (9.4)	0.368
Monthly expenditure in USD, x̄ (SD)	113.1 (55.5)	111.6 (69.4)	95 (53.3)	<0.001
Expenditure on food, %	65.7	70.6	68.8	<0.001
Crowding				
*Not crowded (<5 household members)*	165 (32.3)	175 (34.5)	212 (44.9)	<0.001
*Crowded (≥5 household members)*	346 (67.7)	332 (65.5)	260 (55.1)	
Sanitation				
*Open defecation*	120 (23.5)	69 (13.7)	100 (21.3)	<0.001
*Flush/latrine*	390 (76.5)	436 (86.3)	370 (78.7)	
Drinking water source║				
*Unprotected*	209 (41.4)	163 (32.3)	205 (43.5)	0.001
*Protected*	299 (58.9)	341 (67.7)	266 (56.5)	

HAZ – height for age Z-score, MUAC – mid-upper arm circumference, SD – standard deviation, WAZ – weight for age Z-score, WHZ – weight for height Z-score, x̄ – mean

*Presented as n (%) unless specified otherwise.

†N is number of children aged <5 y.

‡Questions on breastfeeding, vaccination, childhood illnesses and dietary diversity were only asked to the youngest child in the household.

§Minimum Dietary Diversity for the Child (MDD-C).

¶Reduced Copying Strategies Index (rCSI). Higher scores mean frequent use of harmful coping strategies and hence increased food insecurity.

‖Protected water source: piped/tap water and borehole. Unprotected water source: well, rainwater, tanker truck, rain, and surface water.

Overall, study arms were balanced in key maternal and child characteristics. Child wasting was approximately 15% at baseline, with no statistically significant differences across arms. Children in arm 3 had slightly higher stunting rates but fewer recent illnesses. Mothers’ average age (32–34 years) and MUAC (26–27 cm) showed minor variation across arms.

Household crowding was lower in arm 3, with fewer children and fewer total household members, and food security indicators were better than in other arms. Average monthly expenditures were lower in arm 3 (USD 95) than in the other arms (USD 112–113), and all households spent about 70.0% of their budgets on food.

Most households had access to flush or latrine toilets (77.0–86.0%; highest in arm 2), and 57.0–68.0% had access to protected water sources (also highest in arm 2) (Table S8 in the **Online Supplementary Document**).

Across the six-month follow-up period, the study experienced 22.0% attrition overall; this varied slightly across arms (18.0–27.0%) (**Figure 1**). To assess potential attrition bias, we compared baseline characteristics of children and households who were retained *vs.* those lost to follow-up at both midline and endline (Table S9 in the **Online Supplementary Document**). At midline, 287 children (15.2%) were lost to follow-up, while 421 children (22.2%) were lost by endline. Children lost to follow-up did not differ significantly from those retained in key demographic or nutritional characteristics, including child age, sex, weight, height, MUAC, anthropometric Z-scores (WHZ, WAZ, HAZ), wasting prevalence, underweight prevalence, or household food insecurity indicators at baseline (*P* > 0.05). These findings suggest that attrition was likely random and did not introduce systematic bias into our results

### Maternal and child outcomes

**Figure 2:**
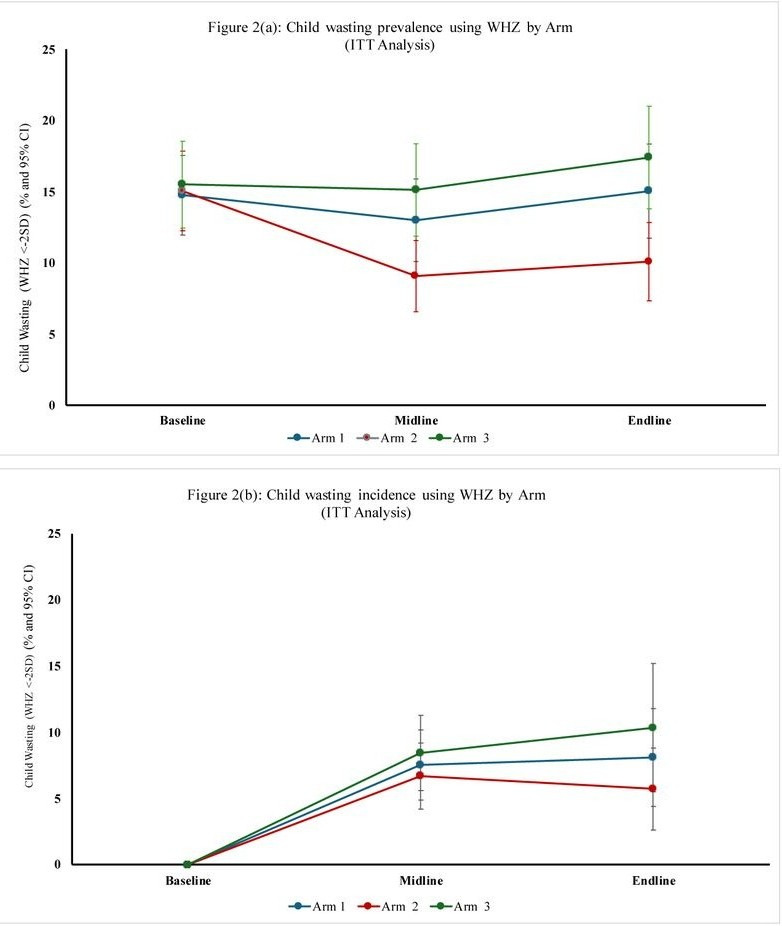
Child wasting. **Panel A.** Prevalence over time using WHZ by arm (ITT analysis). **Panel B.** Incidence using WHZ by arm (ITT analysis).

Using ITT analyses, from baseline to midline, child wasting prevalence decreased significantly in arm 2 from 15.0% (95% confidence interval (CI) = 12.4, 18.0) to 9.1% (95% CI = 6.8, 11.8%) but not in arm 1 (14.7% to 13.0%; confidence intervals overlap) or arm 3 (15.5% to 15.1%; confidence intervals overlap) (**Figure 2**, Panel A). Arm 2 wasting prevalence was statistically different from arm 1 (*P* = 0.039) and arm 3 (*P* = 0.003) at midline. By endline, all arms experienced a slight increase in wasting prevalence (15.0% for arm 1, 10.1% for arm 2, and 17.4% for arm 3) from midline levels, but arm 2 was statistically lower compared to other arms (*P* < 0.05) and sustained the greatest reduction overall from baseline to endline. Analysis of wasting incidence outcomes found similar results (**Figure 2**, Panel B). Over time and between-arm differences in child anthropometry (MUAC, WHZ, WAZ, and HAZ) outcomes, overall and by subgroup (Bay *vs.* Hiran regions; child age <2 *vs.* ≥2 years (Tables S10 and S11 in the **Online Supplementary Document**).

Sensitivity analyses comparing ITT and per-protocol findings showed no differences in trends in child and maternal wasting prevalence but a slight underestimation of wasting incidence with per-protocol methods (Figure S2, Panels A and B in the **Online Supplementary Document**). Given these minor differences, we report wasting prevalence using ITT analysis for the remaining results.

Child wasting prevalence differences existed and even amplified after adjusting for baseline covariates to account for potential confounding (**Table 2**). Similar findings were seen for child underweight prevalence differences in arm 2 compared to other arms, while no effect on stunting prevalence change was observed. Crude and adjusted difference-in-differences analyses confirmed these findings, with protective odds ratios for child wasting in arm 2 *vs.* arm 1, but no difference between arms 3 and 1 (Tables S12 and S13 in the **Online Supplementary Document**). Difference-in-differences models for child underweight, stunting, and MUAC-based wasting showed no meaningful differences between arms in either crude or adjusted models (Tables S14 and S15 in the **Online Supplementary Document**).

**Table 2:** Crude and adjusted differences for child wasting outcomes (prevalence analysis using ITT approach)

Outcome variable and study arm	Unadjusted						Partially adjusted*						Fully adjusted†					
	**Endline – baseline**	***P*-value**	**Midline – baseline**	***P*-value**	**Endline – midline**	***P*-value**	**Endline – baseline**	***P*-value**	**Midline – baseline**	***P*-value**	**Endline – midline**	***P*-value**	**Endline – baseline**	***P*-value**	**Midline – baseline**	***P*-value**	**Endline – midline**	***P*-value**
WHZ, x̄ (95% CI)																		
*Arm 1*	0.111 (0.013, 0.210)	0.026	0.165 (0.072, 0.259)	0.001	0.074 (0.012, 0.137)	0.020	0.263 (0.055, 0.472)	0.013	0.449 (0.253, 0.645)	<0.001	0.185 (0.050, 0.321)	0.007	–0.048 (–0.310, 0.215)	0.721	0.257 (0.006, 0.507)	0.044	0.033 (–0.142, 0.208)	0.712
*Arm 2*	0.148 (0.052, 0.243)	0.003	0.242 (0.151, 0.333)	<0.001	0.085 (0.023, 0.147)	0.007	0.372 (0.095, 0.650)	0.008	0.569 (0.309, 0.829)	<0.001	0.227 (0.047, 0.407)	0.013	0.148 (–0.179, 0.474)	0.375	0.467 (0.157, 0.777)	0.003	0.116 (–0.102, 0.334)	0.298
*Arm 3*	–0.010 (–0.110, 0.089)	0.840	0.092 (–0.004, 0.187)	0.059	0.011 (–0.054, 0.075)	0.749	0.318 (–0.089, 0.725)	0.126	0.477 (0.094, 0.860)	0.015	0.200 (–0.068, 0.467)	0.143	0.231 (–0.212, 0.674)	0.307	0.519 (0.098, 0.940)	0.016	0.154 (–0.144, 0.452)	0.310
MUAC in cm, x̄ (95% CI)																		
*Arm 1*	0.443 (0.352, 0.534)	<0.001	0.366 (0.280, 0.452)	<0.001	0.221 (0.166, 0.275)	<0.001	0.641 (0.435, 0.847)	<0.001	0.483 (0.291, 0.676)	<0.001	0.336 (0.216, 0.456)	<0.001	0.517 (0.254, 0.780)	<0.001	0.403 (0.155, 0.652)	0.001	0.271 (0.114, 0.427)	0.001
*Arm 2*	0.380 (0.291, 0.470)	<0.001	0.303 (0.219, 0.387)	<0.001	0.201 (0.147, 0.255)	<0.001	0.729 (0.455, 1.003)	<0.001	0.515 (0.260, 0.770)	<0.001	0.408 (0.248, 0.568)	<0.001	0.578 (0.250, 0.905)	0.001	0.396 (0.088, 0.704)	0.012	0.327 (0.133, 0.522)	0.001
*Arm 3*	0.185 (0.093, 0.278)	<0.001	0.160 (0.072, 0.248)	<0.001	0.106 (0.049, 0.162)	<0.001	0.734 (0.332, 1.136)	<0.001	0.491 (0.115, 0.867)	0.011	0.439 (0.201, 0.677)	<0.001	0.559 (0.114, 1.004)	0.014	0.354 (–0.064, 0.772)	0.097	0.343 (0.077, 0.609)	0.011

CI – confidence interval, MUAC – mid-upper arm circumference, WHZ – weight for height Z-scores, x̄ – mean

*Partially adjusted for cluster, child age, and sex.

†Fully adjusted for cluster, child age, sex, displacement status, wasting, underweight, stunting at baseline, maternal education, HH monthly expenditure at baseline, sanitation, and access to protected water source at baseline.

Similar to the child, across all arms, maternal wasting prevalence decreased from baseline to midline but increased again at endline. Arm 2 had the lowest prevalence at endline of 11.8% (95% CI = 8.8, 15.3) compared to arm 1 (15.5%) and arm 3 (13.6%), though differences were not statistically significant (Figure S4 in the **Online Supplementary Document**). Regarding over-time and between-arm differences for maternal MUAC overall and by subgroup prevalence and incidence, we found no differences across arms in maternal underweight after adjusting for covariates (Tables S10–12 in the **Online Supplementary Document**).

To address baseline differences in region and IDP distribution across arms, we conducted stratified difference-in-differences analyses (Table S15 in the **Online Supplementary Document**). We observed the protective effects of arm 2 (cash plus SBCC) on child wasting in both the Bay region (predominantly IDP) (adjusted odds ratio (aOR) = 0.47, *P* = 0.029) and the Hiran region (predominantly host community) (aOR = 0.52, *P* = 0.071), though not all effects reached statistical significance. In contrast, arm 3 showed no improvement in either region at any time point.

### Cost analysis

**Figure 3:**
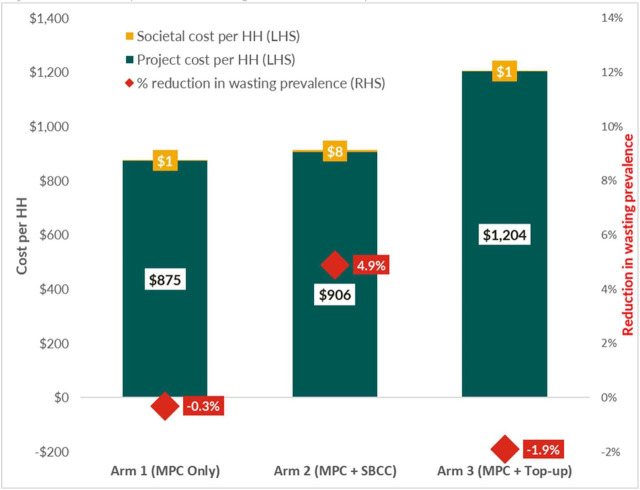
Cost analysis results compared across study arms.

Regarding the cost-efficiency of the intervention, arms 1 and 2 provide the lowest cost per household. The cash transfer ratio (CTR) was lower for arm 3, given the nature of the intervention and the larger cash transfer amount. Given that arm 2 received a comprehensive social and behaviour change communication package, this arm appears to have incurred little additional cost per household over six months (USD 31) compared with the other arms. In terms of overall reductions in wasting prevalence, compared with arm 1, arm 2 had a 4.9% lower prevalence at endline, while arm 3 had a 1.9% higher prevalence. Overall, the CTRs for the three arms, which range from USD 0.69–0.80 of delivery costs per USD 1 in aid, are broadly in line with similar cash interventions in the region. The operational and administrative expenses of the Save the Children International Somalia Country Office and Save the Children International global headquarters – particularly staff salaries – were the main drivers of the CTR across all treatment arms. Direct costs, such as logistics or cash transfer fees, were not major cost drivers. Compared with Hiran, the project had lower delivery costs in Bay, where the transfer value was USD 90, than in Hiran, where it was set at only USD 70 due to the region's lower cost of living (**Figure 3**; Table S16 in the **Online Supplementary Document**).

By endline, there was no statistically significant difference in the change in wasting prevalence between arm 1 and arm 3, and additional costs incurred for ineffective interventions are inherently not cost-effective. Therefore, our overall assessment posits that arm 2 was cost-effective compared to arms 1 and 3. While arm 2 had the greatest societal cost, requiring a beneficiary time investment of USD 8 per household, this investment only accounted for 0.91% of the total cost per household.

### Immediate determinants

At the immediate level, child diet diversity – including the intake of animal-source proteins – improved across all study arms, with arm 3 showing the highest levels by endline. Reports of child illness declined in arms 1 and 2 but increased in arm 3 (**Table 3**; Figure S4 in the **Online Supplementary Document**).

**Table 3:** Household, maternal and child factors related to child wasting by study arm over time

Child wasting drivers and study arm	Baseline, % (95% CI)	Midline, % (95% CI)	Endline, % (95% CI)
**Immediate factors**			
Meet MDD-C			
*Arm 1*	5.1 (2.6, 9.9)	35.0 (29.1, 40.2)	28.1 (23.3, 33.3)
*Arm 2*	12.0 (7.5, 18.8)	33.4 (28.4, 38.9)	32.3 (27.6, 37.1)
*Arm 3*	10.4 (6.1, 17.2)	36.6 (31.4, 42.2)	42.5 (37.3, 47.8)
Child received animal-sourced protein (meat, eggs, milk)			
*Arm 1*	53.1 (45.7, 60.4)	76.2 (71.5, 80.4)	73.5 (68.7, 77.8)
*Arm 2*	58.8 (50.9, 66.2)	74.9 (70.2, 79.0)	73.3 (68.7, 77.4)
*Arm 3*	56.6 (48.3, 64.6)	69.4 (64.6, 73.8)	81.6 (77.4, 85.1)
Had a childhood illness two weeks prior (malaria, diarrhoea, or cough)*			
*Arm 1*	41.1 (34.0, 48.6)	31.5 (26.8, 36.6)	25.4 (21.2, 30.2)
*Arm 2*	31.4 (24.7, 39.1)	31.8 (27.2, 36.7)	27.8 (23.6, 32.4)
*Arm 3*	25.9 (19.3, 33.7)	30.8 (26.4, 35.6)	36.6 (31.9, 41.5)
**Underlying household factors**			
Moderate-severe household hunger scale			
*Arm 1*	48.1 (44.3, 51.9)	32.8 (28.4, 37.5)	44.6 (39.6, 49.8)
*Arm 2*	38.7 (35.1, 43.5)	27.2 (23.2, 31.6)	50.4 (45.5, 55.3)
*Arm 3*	35.5 (31.7, 39.5)	18.5 (15.0, 22.5)	43.7 (38.9, 48.7)
Borderline-poor food consumption score			
*Arm 1*	68.0 (64.4, 71.4)	32.5 (28.1, 37.2)	52.9 (47.7, 58.0)
*Arm 2*	57.0 (53.1, 60.7)	27.6 (23.6, 32.1)	47.1 (42.3, 52.0)
*Arm 3*	50.8 (46.7, 54.9)	34.5 (30.1, 39.2)	38.6 (33.9, 43.6)
Reduced coping strategies index score, x̄ (95% CI)			
*Arm 1*	14.5 (13.7, 15.3)	13.5 (12.5, 14.5)	14.5 (13.5, 15.5)
*Arm 2*	15.3 (14.5, 16.0)	14.3 (13.2, 15.3)	14.6 (13.5, 15.7)
*Arm 3*	14.9 (14.0, 15.7)	12.4 (11.5, 13.3)	14.1 (13.1, 15.0)
Received any childhood vaccination			
*Arm 1*	50.9 (43.4, 58.2)	62.7 (57.5, 67.7)	79.8 (75.4, 83.7)
*Arm 2*	48.4 (40.7, 56.2)	59.4 (54.3, 64.2)	86.8 (83.0, 89.7)
*Arm 3*	46.2 (38.1, 54.4)	67.6 (62.8, 72.1)	75.4 (70.9, 79.5)
Crowded household (≥5 household members)			
*Arm 1*	67.7 (63.5, 71.6)	57.6 (52.7, 62.3)	100
*Arm 2*	65.5 (61.2, 69.5)	59.9 (55.1, 64.4)	99.5 (98.0, 99.9)
*Arm 3*	55.1 (50.6, 59.5)	39.3 (34.7, 44.1)	100
Households practicing open defecation			
*Arm 1*	23.5 (20.0, 27.4)	28.7 (24.1, 33.8)	22.7 (18.7, 27.3)
*Arm 2*	13.7 (10.9, 17.0)	15.0 (11.7, 19.0)	19.0 (15.4, 23.2)
*Arm 3*	21.3 (17.8, 25.2)	27.3 (23.1, 32.0)	33.5 (29.0, 38.4)
Households using unprotected drinking water source			
*Arm 1*	41.1 (36.9, 45.5)	45.1 (39.9, 50.4)	30.0 (25.5, 35.0)
*Arm 2*	32.3 (28.4, 36.6)	34.1 (29.5, 39.1)	24.3 (20.3, 28.8)
*Arm 3*	43.5 (39.1, 48.1)	31.0 (26.6, 35.8)	27.2 (23.0, 31.9)
No access to handwashing facilities			
*Arm 1*	95.5 (93.3, 97.0)	97.7 (95.5, 98.9)	78.8 (74.2, 82.7)
*Arm 2*	99.0 (97.6, 99.6)	94.4 (91.5, 96.3)	83.3 (79.3, 86.6)
*Arm 3*	98.7 (97.2, 99.4)	99.2 (97.6, 99.8)	89.8 (86.3, 92.4)
Correct breastfeeding initiation knowledge (within one hour)			
*Arm 1*	NA*	88.9 (85.0, 91.8)	98.6 (96.7, 99.4)
*Arm 2*	NA*	93.5 (90.4, 95.6)	99.5 (98.0, 99.9)
*Arm 3*	NA*	96.6 (94.3, 98.0)	96.6 (94.3, 98.0)
Correct breastfeeding initiation practice (within one hour)			
Arm 1	NA*	89.2 (85.44, 92.1)	98.9 (97.1, 99.6)
Arm 2	NA*	94.2 (91.3, 96.2)	99.3 (97.6, 99.8)
Arm 3	NA*	96.9 (94.6, 98.2)	98.2 (96.3, 99.1)
Exclusive breastfeeding correct knowledge, %			
*Arm 1*	NA*	56.2 (50.9, 61.3)	70.2 (6.2, 74.7)
*Arm 2*	NA*	63.4 (58.3, 68.1)	77.0 (72.6, 80.9)
*Arm 3*	NA*	68.9 (64.1, 73.3)	68.5 (63.8, 73.0)
Exclusive breastfeeding correct practice			
*Arm 1*	NA*	57.6 (52.3, 62.7)	72.0 (67.2, 76.5)
*Arm 2*	NA*	63.1 (58.1, 67.9)	80.3 (76.0, 83.9)
*Arm 3*	NA*	69.7 (64.9, 74.0)	72.9 (68.2, 77.1)
Optimal age for solid foods initiation knowledge (6–8 months)			
*Arm 1*	NA*	56.9 (52.0, 61.6)	35.9 (31.1, 41.0)
*Arm 2*	NA*	59.9 (55.2, 64.4)	59.8 (54.9, 64.5)
*Arm 3*	NA*	58.3 (53.5, 62.9)	44.0 (39.1, 50.0)
Optimal age for solid foods initiation practice (6–8 months)			
*Arm 1*	NA*	31.6 (27.3, 36.3)	38.7 (33.8, 43.8)
*Arm 2*	NA*	35.6 (31.2, 40.2)	58.8 (53.8, 63.5)
*Arm 3*	NA*	35.5 (31.0, 40.2)	45.3 (40.4, 50.2)
Household has ≥2 children age <5 years			
*Arm 1*	31.3 (27.4, 35.5)	17.7 (14.0, 22.0)	29.8 (25.3, 34.8)
*Arm 2*	27.6 (23.9, 31.7)	14.6 (11.4, 18.6)	26.3 (22.2, 30.8)
*Arm 3*	21.0 (17.5, 24.9)	13.1 (10.1, 16.9)	18.4 (14.9, 22.6)
**Basic factors**			
Total monthly household expenditure in USD, x̄ (95% CI)			
*Arm 1*	113.1 (108, 118.0)	158.3 (151.2, 165.4)	155.9 (148.5, 163.2)
*Arm 2*	111.6 (105.5, 117.7)	155.5 (148.9, 162.0)	167.2 (161.6, 172.7)
*Arm 3*	95.0 (90.2, 99.8)	186.4 (178.9, 193.9)	106.8 (103.7, 109.9)
Average percent of monthly household expenditure on food, x̄ (95% CI)			
*Arm 1*	65.7 (63.9, 67.5)	48.1 (46.2, 49.9)	54.1 (52.3, 55.9)
*Arm 2*	70.6 (68.9, 72.2)	51.4 (49.7, 53.1)	55.7 (53.9, 57.4)
*Arm 3*	68.8 (67.2, 70.4)	50.5 (48.9, 52.0)	49.6 (47.9, 51.3)
Mother has no formal education			
*Arm 1*	82.2 (78.6, 85.3)	NA*	72.9 (68.0, 77.2)
*Arm 2*	82.6 (79.0, 85.7)	NA*	67.9 (63.2, 72.3)
*Arm 3*	86.9 (83.5, 89.6)	NA*	68.4 (63.6, 72.8)
Decision-making on income is by mother or jointly			
*Arm 1*	64.4 (60.1, 68.5)	71.3 (66.3, 75.8)	72.6 (67.7, 76.9)
*Arm 2*	64.4 (60.1, 68.5)	76.5 (72.0, 80.6)	76.9 (72.5, 80.1)
*Arm 3*	63.8 (59.3, 68.0)	78.4 (74.0, 82.2)	76.5 (72.1, 80.5)
Decision-making on healthcare is by mother or jointly			
*Arm 1*	68.6 (64.5, 72.5)	71.3 (66.3, 75.8)	81.5 (77.1, 85.2)
*Arm 2*	74.9 (70.9, 78.5)	71.5 (66.7, 75.9)	79.9 (75.7, 83.6)
*Arm 3*	73.3 (69.1, 77.2)	69.9 (65.2, 74.3)	80.2 (75.9, 83.8)

CI – confidence interval, MDD-C – minimum dietary diversity for child, NA – not available, x̄ – mean

*We did not collect these data at baseline.

These quantitative findings on diet and illness are reinforced by qualitative data from focus group discussions. Across all arms, mothers demonstrated a strong understanding of the importance of dietary diversity for child health and nutrition, with many reporting that they used cash assistance to purchase more nutritious foods:

It is used to buy nutritious food for the children, such as bananas, eggs, and soft food that will be of great help to them. – *Mother, Hiraan, arm 1.*

Eating different food is important for the body and the stomach. A change of diet is good – if you keep on eating one kind of food, you won’t get nutrition from it. – *Mother, Hiraan, arm 1.*

Community members in both Bay and Hiraan also expressed a clear understanding of the causes of malnutrition and child illness. They emphasised the importance of a nutritious diet and breastfeeding while acknowledging persistent barriers such as food insecurity and limited access to health services:

Malnutrition can be prevented in the first six months by breastfeeding. – *Husband, Bay, arm 2.*

Malnutrition results from weakness in the body...caused by lack of proper feeding, discontinuation of breastfeeding, and drinking unsafe water. – *Mother, Hiraan, arm 3.*

Yet, there are barriers to feeding the child a diverse diet and concerns about the child's disease contributing to malnutrition. Mothers in all arms were concerned about measles, diarrhoea, and acute watery diarrhoea as major threats. A mother from Hiran reported:

This year there were more diseases such as measles and acute watery diarrhoea...there is a shortage of vaccines for measles. – *Mother, Hiraan, arm 2.*

### Underlying determinants

Moderate/severe hunger and borderline/poor food consumption scores improved in all arms from baseline to midline but rebounded somewhat by endline (**Table 3**). Vaccination coverage increased across all arms, with arm 2 achieving the highest rates by endline.

Access to protected water sources improved, though open defecation persisted and even increased in arm 3. Handwashing facilities became more available, but gains were limited in arm 3. Breastfeeding practices remained strong, with early initiation rates above 90% across arms. Knowledge and practice of exclusive breastfeeding exceeded 50% overall, with the largest increase in arm 2 (up to 80% at endline).

Qualitative data from focus group discussions with mothers and fathers of children aged <5 years echoed these trends and contextualised the barriers to improving household food security (Figure S4 in the **Online Supplementary Document**). Participants frequently cited inflation, limited purchasing power, and the high cost of food as major challenges. Environmental shocks, such as drought, flooding, and locust infestations, as well as blocked roads and supply disruptions, were also mentioned as key constraints:

The cash money is too small. When we go out to look for cereals, it is too expensive, and the farms we used to depend on were destroyed by locusts. – *Mother, Hiraan, arm 1.*

Monitoring of nutritious food in local markets was conducted by Save the Children Somalia at baseline, midline and endline. Data from market monitoring confirmed that food availability varied significantly by region and season, due to both typical supply patterns and access challenges. Market monitoring methods are described in Text S3 in the **Online Supplementary Document****,** and the full market monitoring report may be made available on request from the corresponding author. In the Bay region, cowpeas were unavailable in September (midline), groundnuts were missing in both September and December (endline), and spinach was absent in December. In Hiraan, availability gaps were more extensive: camel meat, eggs, cowpeas, canned oats, Swiss chard, groundnuts, white maize, lentils, spinach, and pumpkin were consistently unavailable in Mataban and Mahas districts. However, Beletweyne district maintained the availability of all tracked commodities.

Focus group discussion participants reflected this variability – some mothers noted market shortages, while others emphasised that food was available but unaffordable:

There is a chance we do not have a suitable market where we can find everything we need. – *Mother, Hiraan, arm 2.*

There are no big challenges. If you have your money, in the market you can peacefully buy anything. – *Mother, Bay, arm 3.*

Although some food prices remained stable or decreased, the cost of key nutritious and staple foods rose notably in both regions over the study period. In Bay, prices increased for vegetable oil, beans, maize, sorghum, banana, Swiss chard, pumpkin, spinach, potatoes, and goat liver. In Hiraan, beans, lentils, sorghum, white rice, goat liver, lime, and onions saw similar inflation. These price hikes reinforced food access barriers voiced in the focus group discussions:

*When the price of food was normal, the money was enough. But when the price increased, we could only get half of the rice and flour, and some oil and milk. One litre of oil is USD 7, and half a litre of rice is about USD 30. It only lasts 15 days, then we have nothing.* – Mother, Bay, arm 3.

Contributing factors identified through market monitoring included seasonal variability, flash flooding in Bay, drought in Hiraan, damaged supply routes, crop failure, increased taxes, and political instability, all of which were echoed by community members:

Rice and spaghetti are mostly used, but our country’s food has not been harvested. The locusts have destroyed [the crops]. – *Mother, Hiraan, arm 1.*

Currently, we are in inflation. The road between Baidoa and Mogadishu is cut off. There is not enough food for us. – *Husband, Bay, arm 3.*

### Basic determinants

Household expenditures rose from baseline to midline, stabilising in arms 1 and 2 (at approximately USD 160), but dropped sharply in arm 3 (from USD 186 to USD 107) (**Table 3**). Spending on food declined from approximately 70% to 50% across arms. Maternal education and household decision-making improved over time with minimal differences between arms. At baseline, spending patterns were consistent across arms, with most expenditures allocated to food followed by hygiene and health; by midline and endline, the share spent on food increased, and new categories (*e.g.* electricity, fuel, transportation) appeared (Figure S5 in the **Online Supplementary Document**). At endline, food and hygiene spending were similar in arms 2 and 3, but arm 3, despite receiving the largest transfer, allocated relatively less to clean water, medical services, and education, and more to clothing, agriculture, and social activities.

These focus group discussions reinforce that the cash assistance was used for food, water, medical needs, school expenses, and other household needs. A few participants used cash resourcefully, such as for entrepreneurial endeavours:

*That cash assistance has been covering some of our basic needs like water expenses, electricity, educational fees, food, and milk for the children, and businessmen and women got revenue and profits from it.* – Mother, Hiraan, arm 2.

Women in arm 2 share their views on the benefits of SBCC, explaining how they learned important skills and lessons related to nutrition and complementary feeding:

Earlier on I used to give only water and sugar but now I learned exclusively breastfeeding in the first six months. – *Mother, Bay, arm 2.*

The people got the messages, they bought nutritious food for their children, and the change was visible. – *Mother, Hiraan, arm 2.*

## DISCUSSION

This cluster-randomised controlled trial provides robust evidence on the effectiveness and cost-effectiveness of a cash plus SBCC intervention in reducing acute malnutrition among children aged <5 years in Somalia. Specifically, the addition of social and behaviour change communication to unconditional cash transfers (arm 2) led to a significant reduction in child wasting compared to unconditional cash transfer alone (arm 1) and UCT plus a top-up cash amount (arm 3). Arm 2 showed a 5.9 percentage-point reduction and a 39.3% relative reduction in child wasting from baseline to midline, with sustained improvements at endline, while arm 3 – despite higher financial input – did not yield better nutritional outcomes. Moreover, arm 2 was the most cost-effective strategy, achieving significant health impacts at a modest additional cost.

These findings are critical in the Somali context, where humanitarian resources are limited, and acute malnutrition remains prevalent. Our results underscore the importance of integrating SBCC with unconditional cash interventions to enhance their impact on nutrition, rather than relying on cash alone or increasing cash amounts without complementary education or support. Further, we propose potential mechanisms of impact (Box S1 in the **Online Supplementary Document**).

### Comparison with previous studies

Our findings contribute to a growing yet still limited body of rigorous evidence on the nutritional impacts of cash-based interventions in humanitarian settings (Text S6 in the **Online Supplementary Document**). While previous studies have demonstrated that cash transfers can improve food security and dietary diversity [63–72], few have measured direct impacts on acute malnutrition using anthropometric indicators such as WHZ. Our results confirm the potential of cash transfers to improve nutrition outcomes, particularly when combined with SBCC – a finding consistent with a quasi-experimental study in Somalia that found positive impacts of cash plus nutrition counselling [73]. However, that study lacked a cash-only comparison group, limiting its ability to attribute effects specifically to SBCC. Our findings also diverge from studies suggesting that higher-value cash transfers can yield greater impacts on nutrition [74–76]. In our context, increasing the cash amount alone (arm 3) did not yield better outcomes and may have introduced complexity or unintended spending behaviours that diluted nutritional benefits (*e.g.* on debt reduction or social activities). This highlights the importance of context-specific programme design and the potential diminishing returns of increasing transfer size without behavioural support.

Our findings contrast with studies suggesting that higher cash transfers inherently lead to better nutrition outcomes, as arm 2 (cash plus SBCC) was both more effective and far more cost-efficient than a higher-value cash transfer alone. This challenges the assumption that ‘more cash equals more impact’ and highlights the diminishing returns of increasing transfer size without addressing behavioural and market constraints [74,75,77,78]. In contrast, the modest cost of SBCC in arm 2 yielded strong returns, even when accounting for opportunity costs, echoing findings from other evaluations in which layering nutrition counselling or SBCC onto cash transfers significantly improved programme efficiency and outcomes [71,77–79].

Our findings add nuance to the existing evidence base on the duration of cash assistance and its impact on nutrition outcomes. They underscore the importance of considering programme duration when evaluating the impact of cash assistance. While short-term transfers can provide immediate relief, they may not allow households to make strategic, longer-term decisions around spending, savings, or investments in nutrition and health. More research is needed to assess how households adjust their financial decisions when cash support is sustained over a longer and more predictable timeframe. While several studies have shown that longer-duration or sustained cash transfers lead to stronger improvements in nutrition [71,74,75,79], our trial observed the greatest reduction in child wasting at three months, particularly in the cash plus SBCC arm, with limited additional gains by six months. This plateau effect diverges from some literature and may reflect contextual challenges in Somalia. Between midline and endline, the study regions experienced significant flooding, price inflation, and market access disruptions [36], among other challenges, all of which likely constrained households' ability to maintain (or further improve) their diets and care practices. These environmental and economic shocks may have masked the potential for further benefit from continued cash assistance. This underscores the importance of embedding adaptive, tailored to the target groups, and context-sensitive approaches in programme design and ongoing research, especially in volatile humanitarian settings.

### Strengths

In this study, we offer several methodological and practical strengths that advance the evidence base on cash-based nutrition interventions in humanitarian settings. First, we employed a rigorous cluster-randomised controlled trial design across multiple sites, allowing for strong causal inference – an improvement over the quasi-experimental designs that dominate this field. Second, the inclusion of three intervention arms (cash only, cash plus SBCC, and cash plus top-up) enabled direct comparison of additive components, helping to isolate the value of behaviour change communication. Third, we integrated cost evidence and societal cost assessment, providing rare but essential insights into value for money and opportunity costs – critical for donor decision-making (Table S17 in the **Online Supplementary Document**). Fourth, outcomes were measured using gold-standard anthropometric indicators such as WHZ and child wasting prevalence and incidence, enhancing comparability with global nutrition targets. Fifth, the study’s real-time market and qualitative monitoring enabled contextual interpretation of the findings in light of environmental shocks such as flooding and inflation, thereby bolstering external validity. Sixth, the SBCC component was designed to be scalable, low-cost, and embedded within existing community structures, offering a practical and replicable model for humanitarian actors seeking effective, locally adaptable solutions. Further research is needed, however, to determine which specific components of SBCC yielded the greatest impact. A key strength of this study was its robust analytical approach, which included both intention-to-treat and per-protocol analyses, as well as assessments of wasting incidence and prevalence. These complementary methods confirmed the consistency of the findings and strengthened confidence in the internal validity of the observed intervention effects. Finally, geographic coverage across both Bay and Hiraan regions strengthens the generalisability of the findings within diverse humanitarian contexts in Somalia.

### Limitations

Several limitations of this analysis exist. While attrition was moderate (approximately 22%), it was not systematically different across arms or associated with baseline nutritional status, reducing potential bias. The intervention was implemented in a dynamic and highly fragile context, with significant flooding, inflation, and supply chain disruptions occurring between midline and endline. These contextual shocks may have constrained household purchasing power and food access, potentially attenuating the nutritional impacts observed in later months. Nonetheless, the study’s adaptive design – featuring ongoing market monitoring, qualitative assessments, and responsive implementation – helped mitigate some of these challenges by allowing for real-time interpretation and programmatic adjustments. Another limitation is that, although maternal nutrition and health behaviours were measured, the study was not powered to detect statistically significant changes in maternal nutrition outcomes; further research on the prevention of maternal wasting should be prioritised. A key limitation of this study is the absence of a study arm that assessed the combined impact of increased cash (top-up) and SBCC. As a result, we are unable to determine the potential synergistic effects of this combination on nutrition outcomes. While this would have been a valuable comparison, resource constraints limited the number of intervention arms we could feasibly implement within the study’s scope. Additionally, variation in SBCC exposure across clusters and potential spillover effects could not be fully accounted for and may have influenced outcomes. Finally, despite rigorous market monitoring, participants were asked to recall market prices and availability for September, posing a risk of recall bias. Though our cost analysis was robust, limitations, such as subjectivity in opportunity costs and the availability of secondary data, remained (Table S17 in the **Online Supplementary Document****).**

### Policy and programmatic implications

Our findings have clear implications for humanitarian policy and programming. First, they provide compelling evidence that cash alone is not enough to improve child nutrition in crisis settings. Behavioural components are essential for converting economic inputs into nutritional gains. Next, the study supports investments in cost-effective, scalable SBCC models and platforms, such as mother-to-mother groups, which can be layered onto existing cash transfer mechanisms with minimal additional cost [80]. Also, donors and implementers should recognise that increasing the size of cash transfers may not yield proportional improvements in nutrition and may, in some cases, divert funds from more effective, behaviourally informed approaches. Particularly in contexts where markets are underdeveloped or volatile, adding cash without improving decision-making support can lead to suboptimal outcomes. Additionally, integrated monitoring of markets, dietary trends, and environmental shocks is essential for dynamically adapting programming. Currently, cash and nutrition programs are often implemented through siloed systems, with limited coordination between actors such as the Somali Cash Consortium and the Nutrition Cluster, as well as across different ministries. This fragmentation weakens the effectiveness of interventions and limits their potential for impact. A key policy recommendation emerging from this research and stakeholder discussions is to strengthen coordination by linking cash and nutrition actors through established platforms like the Scaling Up Nutrition Movement and the Office of the Prime Minister. A dedicated intersectoral working group should be established to facilitate collaboration, align objectives, and improve the coherence of policy and programmatic responses. Finally, adaptive design, as employed in this trial, should be considered a best practice for future evaluations in complex emergencies.

## CONCLUSIONS

This study provides strong evidence from Somalia that cash plus SBCC was associated with lower WHZ-defined wasting than cash alone, at modest additional delivery costs. The addition of SBCC to cash transfers (arm 2) significantly outperformed both cash alone and increased cash, offering both improved and sustained nutritional outcomes and high value for money. These findings make a compelling case for integrated, multi-component humanitarian assistance strategies that combine financial support with education, especially in fragile and food-insecure settings. As humanitarian funding becomes increasingly constrained, such evidence-based approaches are vital for ensuring that scarce resources deliver maximum impact for vulnerable populations.

## Additional material


Online Supplementary Document
