# Supplementary material for: The effectiveness and cost-effectiveness of cash plus interventions to prevent acute malnutrition in Somalia: evidence from an adaptive cluster randomised control trial
Source: J Glob Health. 2026 May 8;16:04111. doi: 10.7189/jogh.16.04111 (PMC13154337; doi:10.7189/jogh.16.04111)
Supplement: Online Supplementary Document [file jogh-16-04111-s001.pdf]

Supplement to: Akseer N, Alier KK, Grounds S, Garretson S, Mohamud S, Khattak Q, Tripaldi M, Loddo F, Mohamoud SA, Mahdi AY, Nur MA, Abdiqadir M, Mitchell E, Kees A, Mahat MB, Gure M, Jibril DI, Gedi D, P'Rajom MO, Omer M, Farah A, Osman MA, Adan AM, Mohamoud FM, Mohamoud AM, Mohamed AA, Abdi AA, Abdulkadir A, Walton S. The Effectiveness and cost-effectiveness of cash plus interventions to prevent acute malnutrition in Somalia: evidence from an adaptive cluster randomised control trial. J Glob Health. 2026;16:04111.

Figure S1. Timeline of relevant shocks affecting the study areas over study period
Table S1. Mobile Messages Received by BHA Program Participants
Text S1. Arm 2 SBCC Intervention Details
Table S2. Trial inclusion and exclusion criteria
Table S3. Outcome Definitions for CU5 and Mothers
Text S2. Data Collection and Measurement Procedures
Table S4. Exposures: Household Survey Indicators and Definitions
Text S3. Data Analyses Methods
Text S4. Focus Group Discussion Data Collection & Analysis Details
Table S5. Names and Roles of Program Staff Consulted for Cost Analyses
Text S5. Cost Analysis Indicators
Table S6. Market Monitoring Data Collection Sites
Table S7. Food Commodities Monitored During Market Monitoring
Table S8. Characteristics of Households, Mother and Children at Baseline, Midline and Endline
Table S9. Comparison of Child and Household Baseline Characteristics of those Lost to follow up vs Retained in Study at Midline and Endline
Table S10. Change in child and maternal anthropometric outcomes for region and age subgroups (Prevalence analysis using PP Approach)
Table S11. Change in child and maternal anthropometric outcomes for region and age subgroups (Incidence analysis using PP Approach)
Figure S2(a). Child wasting outcome by WHZ using different analysis approaches
Figure S2(b). Maternal wasting outcome by MUAC using different analysis approaches
Table S12. Change in child and maternal anthropometric outcomes: WAZ, HAZ, Maternal MUAC (Prevalence analysis using ITT Approach)
Table S13. Crude and adjusted logistic regression showing difference-in-differences effects for child anthropometry outcomes (Prevalence analysis using ITT Approach)
Table S14. Unadjusted difference in differences of child and maternal wasting outcomes (Prevalence analysis using ITT Approach)
Figure S3. Maternal wasting prevalence using MUAC by Arm (ITT analysis)
Table S15. Unadjusted and adjusted difference in differences of child and maternal wasting outcomes by region (Prevalence analysis using ITT Approach)
Table S16. Cost Analysis Indicators Compared Across Study Arms
Figure S4. Qualitative results.

Figure S5. Household Expenditures at Baseline, Midline, and Endline
Text S6. Effectiveness and Cost-Effectiveness of Cash-Based Interventions on Nutrition Outcomes
Table S17. Strengths and Limitations of Cost Analysis Approach
Box S1. Trial Inclusion and Exclusion Criteria

Appendix.

Figure S1. Timeline of relevant shocks affecting the study areas over study period

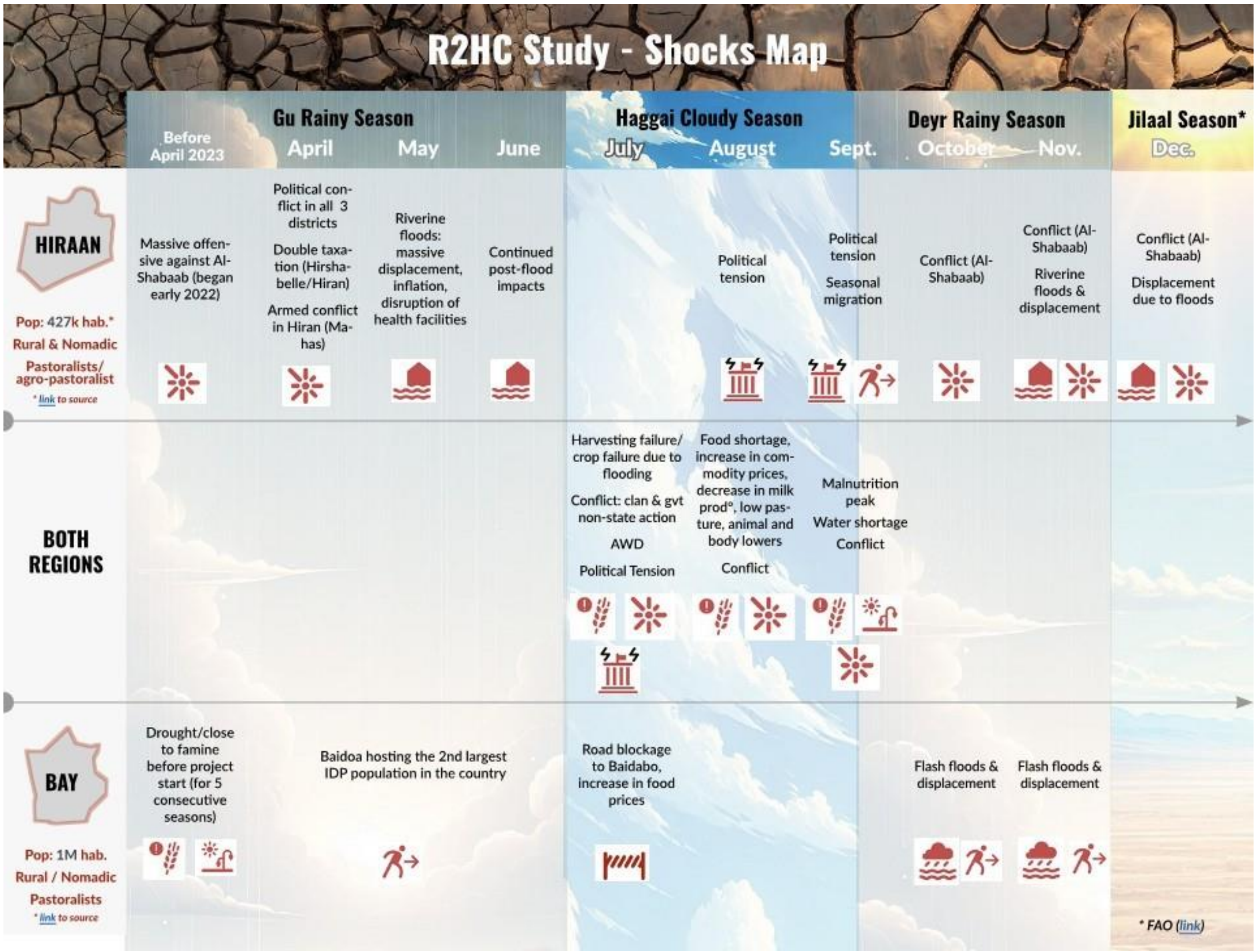

Table S1. Mobile Messages Received by BHA Program Participants

English Translation
Are you aware of the importance of good nutrition for your infants and young children?
We breastfeed our babies exclusively for 6 months because breastmilk is the ideal food for infants and provides all the energy and nutrients that the infant needs for the first months of life.
We breastfeed until our children are two years old because the first two years is our children's opportunity to grow and be ready for the world.
We breastfeed because breastmilk is safe, clean and contains antibodies that help protect against many common childhood illnesses.
Remember to practice good hygiene and sanitation to help prevent illness and ensure your child stays healthy.
Talk to your healthcare provider or community health worker to learn more about IYCF and how you can provide your child with the best possible start in life.
Together, we can give our children the gift of good health and a brighter future. Don't wait, start practicing good Infant and Young Child Feeding practices today.

Text S1. Arm 2 SBCC Intervention Details

SBCC sessions covered the following topics: breastfeeding within the first hour/importance of colostrum; exclusive breastfeeding for 6 months; attachment and positioning of the baby at the breast; breastfeeding misconceptions and myths; complementary feeding (continuing breastfeeding and giving other foods/liquids); medical care; hygiene; common conditions that can affect breastfeeding; physical and mental stimulation of the child; relationships (i) with partner/husband and (ii) with other family members, (e.g. mother-in-law); mother's safety/fear/worries; nutrition of pregnant and lactating mothers.

Save the Children identified 11 staff members to serve as community-based IYCF promoters to train mothers in IYCF practices and engagement strategies for mother-to-mother (M2M) support groups (4 promoters in Bay and 7 in Hiran). SBCC M2M sessions were then led by trained lead mothers who facilitated discussions among 10-15 mothers. Sessions occurred weekly, covering one of the above topics each week (for a total of 12 sessions in 12 weeks), and group members decided on the day of the sessions. Sessions lasted from 45-60 minutes. The SBCC sessions were held in a safe place to enable mothers to exchange ideas, share experiences, give and receive information, and offer and receive support in breastfeeding, child-rearing, and women's health. The lead mother also followed-up with and managed absenteeism during sessions and Save the Children staff through their community-based IYCF promoters provided supportive supervision

In addition to the SBCC sessions, Save the Children organized cooking/food demonstrations for support groups to showcase locally available and affordable nutritious food.

Some mothers enrolled in Arm 2 missed some of the Mother-to-Mother Support Group (MtMSG) sessions, while others migrated due to search of pasture and water for their livestock. The implementation team provided make-up sessions for the mothers who missed some MtMSG sessions. For those who migrated, the implementation team attempted to identify their new locations and collaborated with the local community to arrange MtMSG sessions that were more accessible.

Table S2. Trial inclusion and exclusion criteria

Study Participant	Inclusion Criteria	Exclusion Criteria
**Mothers of children 6 to 59 months**	Enrolled in Save the Children Cash Plus for Nutrition program<br>Have a child that is 6–59 months of age at baseline<br>Subject who is voluntarily willing to participate by signing the consent form	Currently receiving treatment for MAM or SAM<br>Had an episode of severe wasting (SAM) in the past 12 months
**Children 6–59 months**	Enrolled in Save the Children Cash Plus for Nutrition program<br>6–59 months of age at baseline<br>Mother is enrolled in the study	Receiving treatment for MAM or SAM at baseline<br>Had an episode of severe wasting (SAM) in the past 12 months

MAM – Moderate Acute Malnutrition, SAM – Severe Acute Malnutrition

Table S3. Outcome Definitions for CU5 and Mothers

Malnutrition Guidelines for CU5	
Without Acute Malnutrition	MUAC ≥ 125 mm and WHZ[2] ≥ -2 SD,<br>without edema
With Moderate Acute Malnutrition (MAM)	115 mm ≤ MUAC < 124.9 mm<br>and/or -3 SD ≤ WHZ < -2 SD,<br>without edema
With Severe Acute Malnutrition (SAM)	MUAC < 115mm,<br>and/or WHZ < -3 SD,<br>and/or presence of edema
Stunting Guidelines for CU5	
Without Stunting	HAZ > -2 SD
Moderate Stunting	-3 SD < HAZ ≤ -2SD
Severe Stunting	HAZ ≤ -3 SD
Malnutrition Guidelines for Mothers of CU5	
Overweight	MUAC > 300mm
Normal	MUAC ≥ 230 mm
With Moderate Acute Malnutrition (MAM)	MUAC < 230 mm

HAZ – Height-for-age z-score, MUAC – Mid-upper arm circumference, SD – standard deviation, WHZ – Weight-for-height z-score

Text S2. Data Collection and Measurement Procedures

Training for enumerators covered standardized data collection procedures, anthropometric measurement techniques, and tool familiarization. To ensure consistency and data quality, the same team of enumerators was retained throughout the study period, with refresher training sessions conducted before each subsequent data collection phase. Data was collected electronically using KoboToolbox, a secure mobile data collection platform. At baseline, we collected anthropometric measurements for all children under 5 years (CU5) in the household and the mother. We also collected child-level information for the youngest U5 child in the household at baseline, who was then followed longitudinally throughout the study period, regardless of any new births in the household. The survey captured comprehensive household information including socioeconomic indicators, food security metrics, WASH practices, healthcare access, and other factors in alignment with the UNICEF Conceptual Framework on Maternal and Child Nutrition.

Standardized equipment was used for all anthropometric measurements. Height/length measurements were conducted using UNICEF measuring boards with a fixed base and adjustable headpiece, providing precision to 0.1 cm. Children under 24 months were measured in a recumbent position, while those 24 months and older were measured standing. Weight measurements were obtained using SECA 874 battery-operated digital scales, accurate to 0.1 kg. For infants and young children unable to stand independently, weight was measured using the tare function while being held by their mothers. Mid-upper arm circumference (MUAC) was measured using standard non-stretch MUAC tapes with 0.1 cm precision. Quality control measures included daily field supervision and real-time data quality checks. The electronic data collection forms incorporated built-in validation rules to minimize data entry errors.

Table S4. Exposures: Household Survey Indicators and Definitions

**Enabling Causes**	
Context, Capital, and Resources	
Demographic Information and Household Characteristics	Participants were surveyed on a variety of household and demographic characteristics including:<br>– Age of the mother<br>– Sex of the Head of Household<br>– Pregnancy Status of the Mother<br>– Number of CU5 in the household<br>– Displacement due to flooding (endline)<br>– Receipt of Cash Assistance/Livelihood Support from other programs<br><br>Nutrition sensitive/cash programs (cash assistance, food baskets, livelihoods support
Maternal Education	Mothers were asked what their highest level of education was:<br>– No formal education<br>– Madrasa<br>– Primary<br>– Secondary
Maternal Empowerment and Decision-making	Mothers were asked about their involvement in household decision-making in three areas:<br>– Income<br>– Healthcare<br>– Purchases<br>For each area, the decision-maker was either:<br>– Father<br>– Mother<br>– Joint decision-making
Household Assets: Principal Component Analysis	Household assets and wealth were evaluated using the Principal Component Analysis index. The index uses asset indicators rather than income data to visualize long-term economic status.<br><br>The asset index was constructed from these assets and housing characteristics:

	– Livestock ownership<br>– Land ownership (in hectares)<br>– Back account ownership<br>– Household items (electricity, radio, TV, telephone, computer, refrigerator, internet air conditioning)<br>– HH Assets (watch, mobile phone, bicycle, scooter, donkey cart, truck, canoe, tractor, ox plough)<br>– Crowding status<br>– Floor<br>– Roof<br>– Walls<br>– Type of toilet<br>– Water Source<br>Household were then categorized into asset index quintiles: poorest, poorer, middle, richer, and richest.
Household Expenditures	Participants were surveyed on monthly expenditures (absolute dollar value) and expenditure categories.<br><br>Expenditure categories included: food, water, medical needs, agriculture, hygiene, electricity, maternal/child health needs, social activities, transport, communication, clothes and NFIs, debt repayment, fuel, rent, school fees, and savings.
**Underlying Causes**	
Household Food Security	
Household Hunger Scale	The Household Hunger Scale (HHS) measures the scale of households' food deprivation.<br><br>Method: Participants are asked if their HH has experienced any of the following in the past 30 days and how often?<br>– No food to eat of any kind because of lack of resources to get food.<br>– A HH member had to sleep hungry because there was not enough food.

<table>
<tr><td></td><td>– A HH member had to go a whole day and night without eating anything at all because there was not enough food.

Frequencies are coded as rarely (1-2 times), sometimes (3-10 times), and often (>10 times).

Scores are summed and categorized as:
0-1: Little to no hunger
2-3: Moderate hunger
4-6: Severe hunger</td></tr>
<tr><td>Food Consumption Score</td><td>Measures how often household consume food items from the different food groups during a 7-day reference period.

Method: The consumption frequency of each food group is multiplied by its weight.

<table>
<tr><th></th><th>Food Group</th><th>Weight</th></tr>
<tr><td>1</td><td>Main staples</td><td>2</td></tr>
<tr><td>2</td><td>Pulses</td><td>3</td></tr>
<tr><td>3</td><td>Vegetables</td><td>1</td></tr>
<tr><td>4</td><td>Fruit</td><td>1</td></tr>
<tr><td>5</td><td>Meat/Fish</td><td>4</td></tr>
<tr><td>6</td><td>Dairy</td><td>4</td></tr>
<tr><td>7</td><td>Sugar</td><td>0.5</td></tr>
<tr><td>8</td><td>Oils</td><td>0.5</td></tr>
</table>

Scores are summed and categorized as:
0-28: Poor
28-5-42: Borderline
>42: Acceptable

Alternative thresholds for FCS categories were used due to high oil and sugar consumption in Somalia.</td></tr>
<tr><td>Reduced Coping Strategies Index</td><td>Measures the frequency and severity of HH food consumption behaviors due to food shortage in the 7 days prior.

Method: Participants are asked a series of 5 weighted questions and how often they have experienced these in the past 7 days.</td></tr>
</table>

<table>
<tr>
<td></td>
<td>
<table>
<tr><th>Question</th><th>Weight</th></tr>
<tr><td>Relies on less preferred food</td><td>1</td></tr>
<tr><td>Borrows food/or rely on friends &relatives</td><td>2</td></tr>
<tr><td>Limits portion size at mealtimes</td><td>1</td></tr>
<tr><td>Restricts consumption by adults</td><td>3</td></tr>
<tr><td>Reduce the # of meals in a day</td><td>1</td></tr>
</table>

Scores are summed and range from 0-56. Higher scores mean households increasingly use worse coping mechanisms and have increased food insecurity.
</td>
</tr>
<tr>
<td colspan="2">Unhealthy Household Environment</td>
</tr>
<tr>
<td>Household Crowding</td>
<td>Participants provided a household roster at survey timepoints.

Household crowding was defined as:
Not crowded: Less 5 people in the HH
Crowded: 5 or more people in the HH</td>
</tr>
<tr>
<td>Water, Sanitation, and Hygiene Practices (WaSH)</td>
<td>Participants were asked questions about their access to:
– Handwashing facilities with water and soap
– Improved/unimproved sanitation facilities
– Improved/unimproved water sources

Sanitation facilities and water sources were classified as improved or unimproved based on the DHS classification.

Sanitation:
Improved: flushing toilet, pit latrine, composting toilet
Unimproved: open defecation in a bush or field, bucket, other

Water Source:
Improved: piped water, public tap/standpipe, protected well, protected spring, rainwater, tanker truck, cart with small tank</td>
</tr>
</table>

	Unimproved: unprotected well, unprotected spring, surface water, or other
Inadequate Care and Feeding Practices	
Maternal and Child Health	Participants were surveyed on topics related to maternal and child health including place of last birth, whether their last birth was assisted by a skilled-birthing attendant, caesarean birth, and child vaccination history.
Malnutrition Screening	Participants were asked if they or their child received malnutrition screening in a health facility, in the community, or at home in the last 3 months, whether they were referred for malnutrition treatment, and whether their child is currently enrolled in wasting treatment.
SBCC Indicators	Participants were surveyed on topics related to knowledge and practice of health and ICYF behaviors, including initiation and exclusivity of breastfeeding, initiation of liquid and solid foods, water treatment, critical handwashing moments, and awareness of M2M support groups (Arm 2 only).
**Immediate Causes**	
Inadequate Dietary Intake	
Minimum Dietary Diversity – Child	Measures how many of these food groups the child consumed the day before survey data collection:<br>– Breastmilk<br>– Grains, roots, tubers<br>– Legumes, nuts<br>– Dairy products<br>– Flesh foods (meat/fish)<br>– Eggs<br>– Vitamin A rich fruits and vegetables<br>– Other fruits and vegetables<br><br>Scores range from 0 (consumed none of these food groups) to 8 (consumed all of these food

	groups) and dietary diversity is categorized as:<br>– Does not meet dietary diversity: <5 food groups<br>– Meets dietary diversity: 5 or more food groups
Diseases	
Childhood Illness	Participants were asked whether their child experienced diarrhea, cough, or fever, within the past two weeks.

Text S3. Data Analysis Methods

Quantitative Data Management and Statistical Analysis

Data quality checks and validation were conducted by data analysts in near real-time and verified by field teams. Baseline characteristics were summarized using descriptive statistics and we tested for differences across arms. Continuous variables were presented as means and standard deviations, while categorical variables were reported as frequencies and percentages. Main outcomes analyses were based on intent-to-treat prevalence (ITT) approach. We conducted several sensitivity analyses to assess the robustness of our findings, including per protocol (PP) analysis, comparison of prevalence vs incidence analysis, different approaches to handling missing data (complete case analysis vs available case analysis), and different specifications of the time variable (3 months vs 6 months). We also performed a separate analysis for participants lost to follow-up to assess differences from the main cohort. We used multilevel mixed-effects regression models for linear mean change in anthropometric indices and binary outcomes with 95% confidence intervals (CI) and presented results as crude, partially adjusted and fully adjusted for baseline factors for 3 arms at 3 months and 6 months.

We employed a difference-in-differences (DiD) approach using logistic regression to estimate the intervention effects. The DiD compared changes in child and maternal outcomes between intervention arms (arm 2, arm 3) and control arm (arm 1) from baseline to endline, with results expressed as odds ratios (ORs) and CI. We developed three model specifications: an unadjusted model examining only the treatment effect; a partially adjusted model controlling for cluster, child age, sex and wasting at baseline; and a fully adjusted model incorporating time-varying covariates such as maternal factors, household expenditures and WASH indicators. We computed post-estimation statistics including adjusted predictions at the means and average marginal effects for each study arm across time points. Panel effects was confirmed using the Breusch-Pagan Lagrange multiplier test. For comparing multiple intervention arms, we employed the Wald test to assess joint significance, while overall intervention effects were evaluated using likelihood ratio tests. To account for multiple comparisons across the three arms, we applied the Bonferroni correction to maintain the family-wise error rate at $\alpha = 0.05$. All analyses maintained a significance level of $p<0.05$, and results are reported following CONSORT guidelines for cluster randomized trials. Data management and analysis were performed using STATA Version 18 (StataCorp, College Station, TX, USA).

Qualitative Methods

An explanatory sequential approach was taken, collecting qualitative data after the quantitative data in order to further explore and contextualize quantitative findings. Focus group discussions were held with mothers and fathers of CU5 beneficiaries of the BHA program, following a semi-structured guide exploring participants' views on topics such as the BHA program implementation, the use of cash, dietary diversity and food security. A total of 6 focus groups with mothers (one per trial arm per region) and 2 focus groups with husbands (one per region, combining trial arms) were conducted, with 6-8 participants per focus group for a total of 48 mothers and 14 husbands. Focus groups were conducted, recorded, and transcribed in Somali

and then translated into English for analysis. A mix of inductive and deductive approaches were undertaken for analysis. Additional details describing the focus group guide, data collection, and analysis are provided in **Text S4.**

Cost Analysis Methods

The cost-efficiency and cost-effectiveness analyses in this study use a mixed-methods approach to triangulate findings from three retrospective data sources: 1) a desk review, 2) cost estimation and modelling, and 3) consultations with the SC Somalia country office research team.

Data were gathered from project financial reports and focus groups regarding all financial costs of delivering the intervention, including cost of financial procurement (i.e. cash, other SBCC activities, etc.) and implementation costs (i.e. cash delivery costs, training costs, staff salaries, etc.). Quantitative data was analyzed through a retrospective, top-down costing approach: activity-based costing and step-down cost accounting. Data were cleaned and processed, and then cost estimates were confirmed through group and individual consultations with program implementation staff and in-country cash and nutrition experts to include all direct, shared, and 'societal' costs of the intervention (list of staff consulted is available in **Table S5**).

All costs and indicators were calculated in $USD. Cost-efficiency and societal cost analyses were compared across study arms and regions. Societal costs were calculated based on beneficiary time investment for participation in the study activities and program. Further details on the cost data, indicators used, and their definitions can be found in **Text S5**.

Market Monitoring Methods

Market monitoring focused on foods commonly consumed and typical of an energy-based diet as well as fresh and nutritious food items consumed by CU5 and PLW. A questionnaire was developed and administered by trained data collectors to 160 vendors in Bay (n=48) and Hiraan (n=112) in July 2023 and 111 vendors in Bay (n=33) and Hiraan (n=78) in December 2023. The questionnaire asked about the prices of commodities and reasons for changes in pricing. During the December 2023 data collection, recall data was also collected for September 2023 to have three timepoints (July, September, and December) in line with the timing and duration of the trial. Data collectors underwent a one-day training prior to data collection. In addition to interviewing vendors, data collectors also observed markets, took photos, and engaged with stakeholders to assess commodity availability, pricing, and factors affecting these characteristics. A list of the market sites is available in **Table S6**.

The price and availability of foods were reported by district (Baidoa in Bay region and Beledweyne, Mahas, and Mataban in Hiraan region), with foods being categorized into four main types of commodities for an energy-based diet: sentinel foods, fruits and vegetables, cereals, and protein sources. The list of specific food items monitored is provided in **Table S7**.

The lump sum value of commodities was calculated for each district. Changes in market prices over time were analyzed by comparing prices from the three data collection points (July, September, and December).

The market monitoring report is available by request from the corresponding author.

Text S4. Focus Group Discussion Data Collection & Analysis Details

Focus group discussions were held with mothers and fathers of CU5 beneficiaries of the BHA program. The midline survey dataset served as the sampling frame for selecting participants, and mothers were sampled based on education status, age, and head of household status, with the aim of sampling a diverse set of participants. Additionally, participants' distance from their household to the FGD site was also considered for feasibility purposes. The men's focus groups were comprised of the husbands of the mothers who were selected based on this sampling frame and criteria.

Mothers' and husbands' focus group discussion guides were created and explored the following major themes: the BHA program; the use of cash; health-seeking behaviors during pregnancy (or supporting healthy pregnancy for the husbands' focus groups); child sickness and health-seeking behaviors when sick; and dietary diversity and food security. These guides are available in the supplementary materials of the related published study protocol.

A total of 6 mothers' focus group discussions were conducted, with one focus group for each study arm in each region (Bay and Hiraan). One focus group per region was conducted with husbands, combining participants from all three trial arms, for a total of 2 focus groups with husbands. Each focus group was comprised of 6-8 participants, with a total of 48 mothers and 14 husbands participating.

Focus groups were conducted in central community locations in January 2024, about one month after the endline data was collected in December 2023. Trained enumerators confirmed enrollment criteria, obtained informed consent from each participant, and then used the relevant guide to facilitate a structured discussion. Focus groups were conducted, recorded, and transcribed in Somali and were then translated into English for analysis.

Focus group discussions were analyzed through a mix of inductive and deductive approaches following an iterative five-step analytic process. (25) As the focus groups followed a structured guide, the focus group discussion guides informed initial codes, and two study team members then utilized a two-phased approach to develop the codebook, with an initial round of rapid in vivo coding of a designated transcript, followed by a second round of coding to create additional codes. A structured codebook detailing each code was developed and shared with the qualitative data analysis team. (26) ATLAS.ti v. 24 software was used for coding, and additional codes were added during the coding process as necessary. Each transcript was coded by 2-3 team members, and versions of coded transcripts were merged to generate one final coded transcript for each focus group. Reports for each code were exported from Atlas. Both exploratory analysis and explanatory sequential analysis approaches were undertaken, using the qualitative data to attempt to contextualize and understand quantitative findings. (27) Qualitative findings were summarized and organized according to levels of the UNICEF conceptual framework, and data triangulation was achieved by comparing and contrasting findings from both the mothers' and husbands' focus groups with the quantitative findings. (28)

Table S5. Names and roles of stakeholders and program staff consulted for cost analyses

Name	Role	Organization
Adan Yusuf Mahdi	Senior Nutrition TA	SC Somalia
Abdullahi Arays	Head of REALM	SC Somalia
Said Gaafaa	Research Advisor	SC Somalia
Meftuh Omer	Head of Child Survival	SC Somalia
Muhamud Ali Nur	Area MEAL Manager	SC Somalia
Maimun Gure	Nutrition PM	SC Somalia
Dahir Gedi	Food Security and Livelihoods PM	SC Somalia
Sadiq Abdikadir	Area Research Coordinator	SC Somalia
Adam Faradhuub	Research Manager	SC Somalia
Mohamed Billow Mahat	Nutrition Focal Point	SC Somalia
Nimo Hussein Guleid	Nutrition Program Officer	SC Somalia
Simon Fuchs	Lead Economic Advisor	SC International

Text S5. Cost Analysis Indicators

Cost Efficiency

To evaluate cost-efficiency of the intervention, three key indicators were used:

1) Cost Per Household: the total amount of resources spent per beneficiary HH over the program duration.
2) Cash Transfer Ratio (CTR): the amount of non-cash resources spent per $1 of cash transferred to HHs.
3) Operational Efficiency: the amount of resources spent to reach each beneficiary HH per month, irrespective of cash transfer amount.

Cost-Effectiveness

Incremental Cost-Effectiveness Ratio (ICER): the additional cost per beneficiary HH of Arm 2 and Arm 3, each relative to Arm 1 (control arm) to achieve each % increase in non-wasting prevalence (measured by WHZ). This is a ratio of incremental cost (cost per household indicator) to incremental effectiveness (% change in non-WHZ-wasted children).

The study considered the outcome % increase in non-wasting prevalence (measured by WHZ) as the main indicator. Given Arms 1 and 3 did not achieve positive, significant impact on the outcome, the study was able to posit that Arm 2 is cost-effective compared to Arms 1 and 3. However, whether the intervention is overall cost-effectiveness in the local context, which could be measured by comparing Incremental Cost-Effectiveness Ratio (ICER) to a suitable cost-effectiveness threshold, was not established since Arm 3 did not yield any benefit in wasting.

Societal Costs

To evaluate the required beneficiary time investment for participating in the program, societal costs were analyzed and costed using the 'average income per capita in Somalia'[3]:

Arms 1 and 3 Time Investment: the travel and participation time required for registration and nutrition sensitization sessions for all beneficiary HHs.

Arm 2 Time Investment: travel time and participation time of lead and non-lead mothers were estimated for all M2M groups, lead mother training, cooking demonstrations, and individual counseling sessions, as well as the cash transfer related registration requirements included for Arm 1 and 3.

Table S6. Market Monitoring Data Collection Sites

Region	Market Monitoring Data Collection Areas
Bay	Barxada<br>Hanano<br>Ondon<br>Adaada<br>Dugandug<br>Baidoa
Hiraan	Yoobsan/daraawiish<br>Jawil<br>Mahas<br>Gerjiir<br>Beegadid<br>Mataban

Table S7. Food Commodities Monitored During Market Monitoring

**Category**	**Foods**
Sentinel Foods	Sorghum flour, white rice, pasta, wheat flour, imported rice, vegetable oil
Fruits and Vegetables	Banana, Swiss chard, groundnuts, pumpkin, spinach, potatoes, lime, onion
Cereals	Beans, lentils, maize, whaite maize, sorghum, red sorghum, cowpeas, baits (canned)
Protein Sources	Meat, eggs, cooked eggs, goat liver, canned sardines, canned milk, camel meat, camel milk

Table S8: Characteristics of Households, Mother and Children at Baseline, Midline and Endline

Characteristics *	Baseline				Midline				Endline			
	Arm 1 (Cash Only) (n=672)	Arm 2 (Cash Plus SBCC) (n=653)	Arm 3 (Cash Plus Cash top-up) (n=569) (n=569)	p-value new analysis	Arm 1 (Cash Only) (n=544)	Arm 2 (Cash Plus SBCC) (n=563)	Arm 3 (Cash Plus Cash top-up) (n=500)	p-value new analysis	Arm 1 (Cash Only) (n=489)	Arm 2 (Cash Plus SBCC) (n=517)	Arm 3 (Cash Plus Cash top-up) (n=467)	p-value
Region												
Bay	252 (37.5%)	250 (38.3%)	295 (51.8%)	<0.001	185 (34.0%)	207 (36.8%)	263 (52.6%)	<0.001	174 (35.6%)	204 (39.5%)	251 (53.7%)	<0.001
Hiran	420 (62.5%)	403 (61.7%)	274 (48.2%)		359 (66.0%)	356 (63.2%)	237 (47.4%)		315 (64.4%)	313 (60.5%)	216 (46.3%)	
Displacement status												
Host	416 (61.9%)	403 (61.7%)	271 (47.7%)	<0.001	N/A‡	N/A‡	N/A‡		N/A‡	N/A‡	N/A‡	N/A‡
IDP	256 (38.1%)	250 (38.3%)	297 (52.3%)		N/A‡	N/A‡	N/A‡		N/A‡	N/A‡	N/A‡	
Child Characteristics												
Child Sex												
Male	357 (53.1%)	318 (48.7%)	288 (50.6%)	0.271	283 (52.0%)	269 (47.8%)	253 (50.6%)	0.356	260 (53.2%)	244 (47.2%)	235 (50.3%)	0.166
Female	315 (46.9%)	335 (51.3%)	281 (49.4%)		261 (48.0%)	294 (52.2%)	247 (49.4%)		229 (46.8%)	273 (52.8%)	232 (49.7%)	
Child Age,months	34.4 (14.6)	34.7 (14.3)	33.7 (13.9)	0.507	37.4 (14.4)	37.5 (14.2)	36.7 (13.8)	0.569	40.1 (13.7)	40.0 (13.8)	39.5 (13.5)	0.761
Child MUAC	14.3 (1.0)	14.5 (1.0)	14.3 (1.2)	0.069	14.7 (1.1)	14.7 (1.1)	14.5 (1.1)	0.004	14.8 (1.1)	14.8 (1.1)	14.5 (1.1)	<0.001
WHZ-Score	-0.9 (1.1)	-0.9 (1.0)	-0.9 (1.1)	0.963	-0.7 (1.1)	-0.7 (1.1)	-0.8 (1.2)	0.306	-0.8 (1.1)	-0.8 (1.1)	-0.9 (1.1)	0.020
WAZ-Score	-1.2 (0.9)	-1.1 (0.9)	-1.3 (0.9)	0.047	-0.9 (1.0)	-0.8 (1.0)	-1.1 (1.0)	<0.001	-1.0 (1.0)	-0.9 (1.0)	-1.2 (1.1)	<0.001
HAZ-Score	-1.0 (1.3)	-1.0 (1.3)	-1.2 (1.4)	0.007	-0.8 (1.3)	-0.7 (1.4)	-1.1 (1.5)	<0.001	-0.9 (1.4)	-0.7 (1.4)	-1.1 (1.6)	0.004

Wasting (WHZ)												
WHZ ≥ -2 SD	567 (85.3%)	554 (85.0%)	480 (84.5%)	0.933	469 (87.0%)	501 (91.0%)	421 (84.9%)	0.010	407 (85.0%)	446 (89.9%)	380 (82.6%)	0.004
WHZ <-2 SD	98 (14.7%)	98 (15.0%)	88 (15.5%)		70 (13.0%)	50 (9.1%)	75 (15.1%)		72 (15.0%)	50 (10.1%)	80 (17.4%)	
Wasting (MUAC)												
MUAC ≥ 11.5 cm	670 (99.7%)	652 (100.0%)	567 (99.6%)	0.342	535 (99.3%)	546 (98.9%)	488 (98.2%)	0.275	475 (98.7%)	491 (98.8%)	449 (97.6%)	0.26
MUAC < 11.5 cm	2 (0.3%)	0 (0.0%)	2 (0.4%)		4 (0.7%)	6 (1.1%)	9 (1.8%)		6 (1.3%)	6 (1.2%)	11 (2.4%)	
Underweight (WAZ)												
WAZ ≥ -2 SD	554 (82.7%)	556 (85.1%)	455 (80.0%)	0.058	471 (87.6%)	491 (89.0%)	411 (82.7%)	0.009	409 (85.0%)	444 (89.3%)	359 (78.0%)	<0.001
WAZ <-2 SD	116 (17.3%)	97 (14.9%)	114 (20.0%)		67 (12.4%)	61 (11.0%)	86 (17.3%)		72 (15.0%)	53 (10.7%)	101 (22.0%)	
Stunting (HAZ)												
HAZ ≥ -2 SD	537 (80.4%)	534 (82.0%)	410 (72.3%)	<0.001	451 (84.1%)	463 (84.2%)	359 (72.5%)	<0.001	387 (81.1%)	415 (84.0%)	327 (72.5%)	<0.001
HAZ <-2 SD	131 (19.6%)	117 (18.0%)	157 (27.7%)		85 (15.9%)	87 (15.82%)	136 (27.5%)		90 (18.9%)	79 (16.0%)	130 (28.5%)	
Youngest child ever breastfed †												
No	18 (10.3%)	27 (16.9%)	18 (12.6%)	0.198	78 (19.2%)	60 (13.8%)	87 (20.9%)	0.020	N/A‡	N/A‡	N/A‡	N/A‡
Yes	157 (89.7%)	133 (83.12%)	125 (87.4%)		328 (80.8%)	374 (86.2%)	330 (79.1%)		N/A‡	N/A‡	N/A‡	
Received any childhood vaccination †												
No	86 (49.1%)	82 (51.6%)	77 (53.8%)	0.704	154 (37.9%)	176 (40.6%)	138 (33.1%)	0.075	73 (20.2%)	53 (13.2%)	96 (24.6%)	<0.001
Yes	89 (50.9%)	77 (48.4%)	66 (46.2%)		252 (62.1%)	258 (59.4%)	279 (66.9%)		289 (79.8%)	347 (86.8%)	295 (75.4%)	
Had childhood illness 2 weeks prior ¶ †												

No	103 (58.9%)	109 (68.6%)	106 (74.1%)	0.013	277 (68.2%)	297 (68.4%)	294 (70.5%)	0.734	270 (74.6%)	289 (72.2%)	248 (63.4%)	0.002
Yes	72 (41.1%)	50 (31.4%)	37 (25.9%)		129 (31.8%)	137 (31.6%)	123 (29.5%)		92 (25.4%)	111 (27.8%)	143 (36.6%)	
Child Minimum Dietary Diversity (MDD-C) †												
Do Not Meet MDD	149 (94.9%)	117 (88.0%)	112 (89.6%)	0.093	217 (66.4%)	248 (66.8%)	207 (63.5%)	0.613	223 (71.9%)	253 (67.8%)	195 (57.5%)	<0.001
Meet MDD	8 (5.1%)	16 (12.0%)	13 (10.4%)		110 (33.6%)	123 (33.2%)	119 (36.5%)		87 (28.1%)	120 (32.2%)	144 (42.5%)	
Animal-sourced protein (meat, eggs, milk)												
No animal-sourced protein	82 (46.9%)	66 (41.3%)	62 (43.4%)	0.579	98 (24.1%)	106 (24.4%)	127 (30.5%)	0.065	96 (26.5%)	107 (26.7%)	72 (18.4%)	
At least one animal-sourced protein	93 (53.1%)	94 (58.7%)	81 (56.6%)		308 (75.9%)	328 (75.6%)	290 (69.5%)		266 (73.5%)	293 (73.3%)	319 (81.6%)	
Mother Characteristics												
Age, years	32.2 (9.0)	34 (10.8)	32.9 (9.9)	0.014	N/A‡	N/A‡	N/A‡	N/A‡	32.4 (9.1)	34.2 (11.2)	32.8 (9.7)	0.024
Age (Dichotomized)												
18-34 Yrs	306 (59.9%)	281 (55.5%)	278 (59.2%)	0.325	N/A‡	N/A‡	N/A‡	N/A‡	219 (60.5%)	216 (54.0%)	230 (59.1%)	0.155
35+ Yrs	205 (40.1%)	225 (44.5%)	192 (40.8%)		N/A‡	N/A‡	N/A‡		143 (39.5%)	184 (46.0%)	159 (40.9%)	
MUAC, cm	26.7 (4.0)	27.0 (3.8)	26.1 (3.6)	0.002	27.9 (4.4)	27.8 (3.9)	26.6 (3.5)	<0.001	27.3 (4.5)	27.6 (4.0)	26.7 (3.5)	0.006
Maternal Wasting (MUAC)												
MUAC < 23 cm	67 (13.9%)	55 (11.5%)	69 (15.5%)	0.193	316 (91.6%)	321 (89.4%)	342 (89.5%)	0.550	306 (84.5%)	353 (88.3%)	338 (86.5%)	0.325
MUAC ≥ 23 cm	415 (86.1%)	424 (88.5%)	375 (84.5%)		29 (8.4%)	38 (10.6%)	40 (10.5%)		56 (15.%)	47 (11.7%)	53 (13.5%)	
Pregnancy status												
No	420 (82.2%)	400 (79.1%)	370 (78.4%)	0.277	297 (85.1%)	321 (85.8%)	314 (80.7%)	0.117	315 (87.0%)	352 (88.2%)	324 (83.1%)	0.094

Yes	91 (17.8%)	106 (20.9%)	102 (21.6%)		52 (14.9%)	53 (14.2%)	75 (19.3%)		47 (13.0%)	47 (11.8%)	66 (16.9%)	
Education level												
No Formal Education	420 (82.2%)	418 (82.6%)	410 (86.9%)	0.215	N/A‡	N/A‡	N/A‡	N/A‡	263 (72.8%)	271 (67.9%)	266 (68.4%)	0.275
Madrasa	51 (10.0%)	51 (10.1%)	40 (8.5%)		N/A‡	N/A‡	N/A‡		80 (22.2%)	98 (24.6%)	89 (22.9%)	
Primary & Secondary	40 (7.8%)	37 (7.3%)	22 (4.7%)		N/A‡	N/A‡	N/A‡		18 (5.0%)	30 (7.5%)	34 (8.7%)	
Household Characteristics												
Mother head of household												
No	360 (70.5%)	354 (70.0%)	339 (71.8%)	0.804	N/A‡	N/A‡	N/A‡	N/A‡	145 (41.3%)	152 (39.4%)	135 (37.0%)	0.494
Yes	151 (29.5%)	152 (30.0%)	133 (28.2%)		N/A‡	N/A‡	N/A‡		206 (58.7%)	234 (60.6%)	230 (63.0%)	
Decision making on income												
Jointly	215 (42.5%)	205 (41.0%)	199 (42.4%)	0.95	132 (37.9%)	133 (35.9%)	181 (46.5%)	0.005	152 (42.1%)	157 (39.3%)	197 (50.8%)	0.002
Mother	111 (21.9%)	117 (23.4%)	100 (21.3%)		116 (33.3%)	151 (40.7%)	124 (31.9%)		110 (30.5%)	150 (37.6%)	100 (25.8%)	
Father	180 (35.6%)	178 (35.6%)	170 (36.3%)		100 (28.8%)	87 (23.4%)	84 (21.6%)		99 (27.4%)	92 (23.1%)	91 (23.4%)	
Decision making on healthcare												
Jointly	248 (48.9%)	266 (53.1%)	254 (54.2%)	0.188	148 (42.5%)	136 (36.5%)	167 (42.9%)	0.143	192 (53.0%)	193 (48.4%)	215 (55.4%)	0.243
Mother	100 (19.7%)	109 (21.8%)	90 (19.2%)		100 (28.7%)	130 (35.0%)	105 (27.0%)		103 (28.5%)	126 (31.6%)	96 (24.7%)	
Father	159 (31.4%)	126 (25.2%)	125 (26.7%)		100 (28.7%)	106 (28.5%)	117 (30.1%)		67 (18.5%)	80 (20.0%)	77 (19.9%)	
Number of U5 children												
1 child	351 (68.7%)	367 (72.4%)	373 (79.0%)	0.001	289 (82.3%)	321 (85.4%)	338 (86.9%)	0.215	254 (70.2%)	295 (73.8%)	319 (81.6%)	<0.001
2+ children	160 (31.3%)	140 (27.6%)	99 (21.0%)		62 (17.7%)	55 (14.6%)	51 (13.1%)		108 (29.8%)	105 (26.2%)	72 (18.4%)	

Household Hunger Scale (0 best-6 worse)												
Little to no Hunger (≤ 1 score)	283 (55.4%)	307 (60.7%)	307 (65.0%)	0.001	239 (68.5%)	269 (71.9%)	317 (81.5%)	0.001	154 (100.0%)	119 (100.0 %)	158 (100.0 %)	-
Moderate Hunger (2-3 score)	219 (42.9%)	197 (38.9%)	165 (35.0%)		109 (31.2%)	104 (27.8%)	71 (18.2%)		- §	- §	- §	
Severe Hunger (4-6 score)	9 (1.7%)	2 (0.4%)	0 (0.0%)		1 (0.3%)	1 (0.3%)	1 (0.3%)		- §	- §	- §	
Food Consumption Score (adjusted for high oils & sugar consumption)												
Poor (≤ 28 score)	262 (51.3%)	231 (45.6%)	189 (40.0%)	0.007	63 (18.0%)	49 (13.1%)	52 (13.4%)	0.278	120 (33.2%)	128 (32.0%)	93 (23.8%)	0.013
Borderline (28.5-42 score)	124 (24.3%)	131 (25.9%)	125 (26.5%)		120 (34.4%)	134 (35.8%)	149 (38.3%)		112 (30.9%)	125 (31.3%)	119 (30.4%)	
Acceptable (≥ 43 score)	125 (24.4%)	144 (28.5%)	158 (33.5%)		166 (47.6%)	191 (51.1%)	188 (48.3%)		130 (35.9%)	147 (36.7%)	179 (45.8%)	
Coping Strategies Index Score (0 best-56 worse) ‖	14.5 (9.0)	15.3 (8.7)	14.9 (9.4)	0.368	13.5 (9.9)	14.3 (10.7)	12.4 (8.7)	0.033	14.5 (9.8)	14.6 (11.5)	14.1 (9.7)	0.756
Monthly Expenditure, Total (USD)	113.1 (55.5)	111.6 (69.4)	95 (53.3)	<0.001	158 (67.5)	155 (64.5)	186 (75.2)	<0.001	155.9 (70.4)	167.2 (56.4)	147.7 (63.3)	<0.001
Percent of Expenditure on Food (%)	65.7%	70.6%	68.8%	<0.001	48.1%	51.4%	50.5%	0.022	54.1%	55.7%	49.6%	<0.001
Crowding												
Not Crowded (<5 household members)	165 (32.3%)	175 (34.5%)	212 (44.9%)	<0.001	173 (42.4%)	175 (40.1%)	253 (60.7%)	<0.001	0 (0.0%)	2 (0.4%)	0 (0.0%)	0.157
Crowded (≥ 5 household members)	346 (67.7%	332 (65.5%)	260 (55.1%)		235 (57.6%)	261 (59.9%)	164 (39.3%)		362 (100.0%)	398 (99.6%)	391 (100.0 %)	
Sanitation												
Open defecation	120 (23.5%)	69 (13.7%)	100 (21.3%)	<0.001	96 (28.7%)	55 (15.0%)	105 (27.3%)	<0.001	82 (22.7%)	75 (19.0%)	129 (33.5%)	<0.001

Flush/Latrine	390 (76.5%)	436 (86.3%)	370 (78.7%)		239 (71.3%)	312 (85.0%)	279 (72.7%)		279 (77.3%)	319 (81.0%)	256 (66.5%)	
Drinking water source **												
Unprotected Source	209 (41.4%)	163 (32.3%)	205 (43.5%)	0.001	157 (45.1%)	127 (34.1%)	120 (31.0%)	<0.001	108 (30.0%)	97 (24.3%)	106 (27.3%)	0.211
Protected source	299 (58.9%)	341 (67.7%)	266 (56.5%)		191 (54.9%)	245 (65.9%)	267 (69.0%)		252 (70.0%)	302 (75.7%)	72.7%)	

CM – centimeters, HAZ – height for age z-score, IDP – internally displaced person, MUAC – mid-upper arm circumference, SBCC – social behavior change communication, SD – standard deviation, WAZ – weight for age z-score, WHZ – weight for height z-score

*Footnote 1. Response rate for the different variables varied

†Footnote 2. Questions on breastfeeding, vaccination, childhood illnesses and dietary diversity were only asked to the youngest child in the household

‡Footnote 3. N/A: indicate variables were not collected at this time

§Footnote 4. No data point

¶Footnote 5. Minimum Dietary Diversity for the Child

‖Footnote 6. Coping strategies index: A higher score mean more use of worse coping mechanisms and hence increased food insecurity.

**Footnote 7. Protected water source: piped/tap water and birehole; Unprotected water source: well, rainwater, tanker truck, rain, and surface water

Table S9: Comparison of Child and Household Baseline Characteristics of those Lost to follow up vs Retained in Study at Midline and Endline

	Midline			Endline		
	U5 children lost to follow-up n=287	U5 children Retained n=1,607	p-value	U5 children lost to follow-up n=421	U5 children Retained n=1,473	p-value
Child Characteristics						
Child Sex						
Male	158 (55.1%)	805 (50.1%)	0.122	224 (53.2%)	739 (50.2%)	0.272
Female	129 (44.9%)	802 (49.9%)		197 (46.8%)	734 (49.8%)	
Age, month	33.98 (14.10)	34.34 (14.32)	0.694	34.21 (14.21)	34.31 (14.30)	0.898
Weight, kg	11.82 (2.68)	11.85 (2.56)	0.850	11.92 (2.64)	11.83 (2.56)	0.549
Height, cm	88.91 (11.54)	89.66 (11.55)	0.308	89.67 (11.55)	89.51 (11.55)	0.802
MUAC, cm	14.30 (0.98)	14.39 (1.10)	0.214	14.33 (1.01)	14.39 (1.10)	0.368
Z Scores						
WHZ-Score	-0.84 (1.12)	-0.92 (1.09)	0.260	-0.91 (1.16)	-0.91 (1.08)	0.972
WAZ-Score	-1.21 (0.99)	-1.19 (0.91)	0.636	-1.16 (0.98)	-1.20 (0.90)	0.373
HAZ-Score	-1.17 (1.41)	-1.01 (1.32)	0.063	-0.98 (1.38)	-1.05 (1.32)	0.332
Child outcome						
Wasting, WHZ						
Not wasted	251 (87.8%)	1,350 (84.4%)	0.147	356 (84.8%)	1,245 (85.0%)	0.911
Wasted	35 (12.2%)	249 (15.6%)		64 (15.2%)	220 (15.0%)	
Underweight, WAZ						
Normal weight for age	228 (79.4%)	1,337 (83.3%)	0.111	343 (81.5%)	1,222 (83.1%)	0.444
Underweight for age	59 (20.6%)	268 (16.7%)		78 (18.5%)	249 (16.9%)	
Household Food Insecurity Indicators	Household, n=231	Household n=1,259		Household,n=337	Household, n=1,153	
Household Hunger Score						
Little to no Hunger	139 (60.4%)	758 (60.2%)	0.843	204 (60.7%)	693 (60.1%)	0.520
Moderate Hunger	90 (39.1%)	491 (39.0%)		128 (38.1%)	453 (39.3%)	
Severe Hunger	1 (0.4%)	10 (0.8%)		4 (1.2%)	7 (0.6%)	
Food Consumption Score						
Poor	118 (51.3%)	564 (44.8%)	0.165	159 (47.3%)	523 (45.4%)	0.816
Borderline	50 (21.7%)	330 (26.2%)		83 (24.7%)	297 (25.8%)	
Acceptable	62 (27.0%)	365 (29.0%)		94 (28.0%)	333 (28.9%)	
Reduced Consumption Strategies Index (mean)	14.817	14.878	0.925	14.128	15.085	0.087

HAZ – height for age z-score, MUAC – mid-upper arm circumference, WAZ – weight for age z-score, WHZ – weight for height z-score

Table S10. Change in child and maternal anthropometric outcomes for region and age subgroups (Prevalence analysis using PP Approach)

		**Overall**						**By Region**												**By Age**											
								Bay						***Hiran***						***< 2years***						***≥ 2 years***					
**Outcome variable**	**Study Arm**	**Endline - Baseline**	**p-val**	**Midline - Baseline**	**p-val**	**Endline- Midline**	**p-val**	**Endline - Baseline**	**p-val**	**Midline - Baseline**	**p-val**	**Endline- Midline**	**p-val**	**Endline - Baseline**	**p-val**	**Midline - Baseline**	**p-val**	**Endline- Midline**	**p-val**	**Endline - Baseline**	**p-val**	**Midline - Baseline**	**p-val**	**Endline- Midline**	**p-val**	**Endline - Baseline**	**p-val**	**Midline - Baseline**	**p-val**	**Endline- Midline**	**p-val**
**Child Outcomes**																															
WHZ (mean/ 95% CI)	Arm 1	0.111 (0.013, 0.210)	0.026	0.165 (0.072, 0.259)	0.001	0.055 (-0.009, 0.119)	0.093	0.145 (-0.007, 0.298)	0.061	0.259 (0.120, 0.397)	<0.001	0.073 (-0.025, 0.170)	0.143	0.092 (-0.037, 0.221)	0.164	0.112 (-0.015, 0.238)	0.083	0.044 (-0.040, 0.129)	0.305	0.147 (-0.172, 0.465)	0.364	0.324 (-0.002, 0.651)	0.052	0.057 (-0.137, 0.251)	0.566	0.105 (0.003, 0.207)	0.043	0.136 (0.042, 0.231)	0.005	0.054 (-0.012, 0.120)	0.107
	Arm 2	0.148 (0.052, 0.243)	0.003	0.242 (0.151, 0.333)	<0.001	0.080 (0.018, 0.142)	0.012	0.059 (-0.083, 0.201)	0.416	0.301 (0.172, 0.430)	<0.001	0.037 (-0.054, 0.128)	0.422	0.206 (0.077, 0.336)	0.002	0.204 (0.078, 0.329)	0.002	0.108 (0.023, 0.193)	0.013	0.350 (0.025, 0.676)	0.035	0.641 (0.310, 0.972)	<0.001	0.218 (0.027, 0.408)	0.025	0.115 (0.017, 0.213)	0.022	0.177 (0.086, 0.267)	<0.001	0.052 (-0.012, 0.116)	0.113
	Arm 3	-0.010 (-0.110, 0.089)	0.84	0.092 (-0.004, 0.187)	0.059	-0.009 (-0.074, 0.056)	0.796	0.040 (-0.087, 0.167)	0.536	0.160 (0.045, 0.276)	0.007	0.023 (-0.058, 0.105)	0.572	-0.070 (-0.224, 0.084)	0.373	0.011 (-0.140, 0.162)	0.886	-0.046 (-0.148, 0.055)	0.37	0.081 (-0.278, 0.439)	0.658	0.092 (-0.275, 0.458)	0.623	0.064 (-0.141, 0.269)	0.539	-0.023 (-0.124, 0.078)	0.657	0.092 (-0.003, 0.186)	0.056	-0.022 (-0.089, 0.044)	0.513
WAZ (mean/ 95% CI)	Arm 1	0.169 (0.082, 0.257)	<0.001	0.221 (0.144, 0.298)	<0.001	0.087 (0.034, 0.140)	0.001	0.132 (-0.006, 0.269)	0.061	0.198 (0.081, 0.315)	0.001	0.065 (-0.016, 0.146)	0.118	0.191 (0.078, 0.304)	0.001	0.234 (0.133, 0.335)	<0.001	0.100 (0.030, 0.170)	0.005	0.062 (-0.224, 0.348)	0.669	0.264 (-0.015, 0.543)	0.064	0.055 (-0.115, 0.225)	0.522	0.188 (0.098, 0.278)	<0.001	0.213 (0.138, 0.289)	<0.001	0.094 (0.040, 0.147)	0.001
	Arm 2	0.233 (0.148, 0.319)	<0.001	0.310 (0.235, 0.384)	<0.001	0.119 (0.067, 0.170)	<0.001	0.225 (0.096, 0.354)	0.001	0.370 (0.261, 0.478)	<0.001	0.123 (0.047, 0.199)	0.001	0.239 (0.124, 0.353)	<0.001	0.270 (0.169, 0.371)	<0.001	0.116 (0.046, 0.186)	0.001	0.380 (0.088, 0.672)	0.011	0.697 (0.415, 0.980)	<0.001	0.196 (0.029, 0.363)	0.021	0.210 (0.122, 0.297)	<0.001	0.247 (0.174, 0.319)	<0.001	0.103 (0.051, 0.155)	<0.001
	Arm 3	0.074 (-0.015, 0.163)	0.103	0.143 (0.066, 0.221)	<0.001	0.039 (-0.015, 0.093)	0.155	0.129 (0.014, 0.244)	0.028	0.213 (0.115, 0.310)	<0.001	0.065 (-0.003, 0.132)	0.062	0.008 (-0.128, 0.144)	0.903	0.063 (-0.058, 0.184)	0.308	0.009 (-0.075, 0.092)	0.838	0.180 (-0.142, 0.502)	0.272	0.293 (-0.020, 0.606)	0.067	0.125 (-0.054, 0.305)	0.171	0.059 (-0.031, 0.149)	0.200	0.122 (0.046, 0.198)	0.002	0.023 (-0.031, 0.077)	0.409
HAZ (mean/ 95% CI)	Arm 1	0.120 (-0.004, 0.244)	0.059	0.135 (0.024, 0.246)	0.017	0.062 (-0.012, 0.135)	0.099	0.013 (-0.182, 0.208)	0.895	0.021 (-0.162, 0.204)	0.82	-0.003 (-0.119, 0.113)	0.962	0.181 (0.019, 0.343)	0.029	0.200 (0.060, 0.339)	0.005	0.099 (0.004, 0.195)	0.042	-0.075 (-0.517, 0.367)	0.738	-0.057 (-0.422, 0.307)	0.756	-0.030 (-0.256, 0.195)	0.791	0.154 (0.029, 0.279)	0.015	0.169 (0.055, 0.283)	0.004	0.081 (0.005, 0.157)	0.036
	Arm 2	0.250 (0.129, 0.371)	<0.001	0.241 (0.134, 0.348)	<0.001	0.120 (0.049, 0.192)	0.001	0.324 (0.143, 0.505)	<0.001	0.291 (0.121, 0.461)	0.001	0.169 (0.061, 0.277)	0.002	0.200 (0.037, 0.363)	0.016	0.209 (0.070, 0.347)	0.003	0.088 (-0.008, 0.184)	0.071	0.064 (-0.391, 0.519)	0.781	0.403 (0.034, 0.773)	0.032	0.002 (-0.220, 0.224)	0.987	0.279 (0.158, 0.400)	<0.001	0.215 (0.106, 0.325)	<0.001	0.144 (0.071, 0.218)	<0.001
	Arm 3	0.185 (0.059, 0.312)	0.004	0.120 (0.008, 0.233)	0.036	0.093 (0.018, 0.168)	0.015	0.215 (0.053, 0.378)	0.009	0.161 (0.009, 0.314)	0.038	0.105 (0.008, 0.202)	0.034	0.149 (-0.045, 0.344)	0.133	0.073 (-0.094, 0.239)	0.391	0.079 (-0.035, 0.193)	0.175	0.126 (-0.371, 0.623)	0.618	0.170 (-0.243, 0.583)	0.417	0.080 (-0.160, 0.321)	0.512	0.193 (0.068, 0.319)	0.002	0.114 (-0.001, 0.228)	0.051	0.096 (0.019, 0.172)	0.014
MUAC (mean/ 95% CI)	Arm 1	0.443 (0.352, 0.534)	<0.001	0.366 (0.280, 0.452)	<0.001	0.223 (0.167, 0.279)	<0.001	0.508 (0.360, 0.656)	<0.001	0.354 (0.212, 0.496)	<0.001	0.254 (0.163, 0.345)	<0.001	0.405 (0.290, 0.521)	<0.001	0.373 (0.265, 0.482)	<0.001	0.206 (0.134, 0.277)	<0.001	0.549 (0.305, 0.792)	<0.001	0.441 (0.220, 0.661)	<0.001	0.239 (0.091, 0.387)	0.002	0.424 (0.326, 0.522)	<0.001	0.353 (0.259, 0.446)	<0.001	0.220 (0.159, 0.280)	<0.001
	Arm 2	0.380 (0.291, 0.470)	<0.001	0.303 (0.219, 0.387)	<0.001	0.200 (0.145, 0.255)	<0.001	0.271 (0.132, 0.410)	<0.001	0.315 (0.182, 0.447)	<0.001	0.131 (0.046, 0.216)	0.003	0.453 (0.337, 0.570)	<0.001	0.296 (0.187, 0.404)	<0.001	0.246 (0.174, 0.317)	<0.001	0.446 (0.195, 0.696)	0.001	0.485 (0.259, 0.710)	<0.001	0.254 (0.108, 0.400)	0.001	0.370 (0.274, 0.466)	<0.001	0.274 (0.184, 0.364)	<0.001	0.189 (0.130, 0.248)	<0.001
	Arm 3	0.185 (0.093, 0.278)	<0.001	0.160 (0.072, 0.248)	<0.001	0.094 (0.037, 0.151)	0.001	0.136 (0.012, 0.260)	0.031	0.080 (-0.039, 0.199)	0.185	0.068 (-0.009, 0.144)	0.083	0.243 (0.105, 0.382)	0.001	0.253 (0.123, 0.383)	<0.001	0.125 (0.040, 0.210)	0.004	0.254 (-0.023, 0.530)	0.072	0.038 (-0.211, 0.287)	0.765	0.147 (-0.010, 0.304)	0.067	0.176 (0.077, 0.274)	<0.001	0.178 (0.084, 0.272)	<0.001	0.084 (0.023, 0.145)	0.007
**Maternal Outcome**																															
Maternal MUAC (mean/ 95% CI)	Arm 1	0.665 (0.227, 1.104)	0.003	1.290 (0.887, 1.693)	<0.001	0.357 (0.081, 0.633)	0.011	1.248 (0.605, 1.891)	<0.001	2.106 (1.530, 2.682)	<0.001	0.553 (0.173, 0.934)	0.004	0.249 (-0.350, 0.849)	0.414	0.709 (0.152, 1.266)	0.013	0.204 (-0.192, 0.600)	0.313	0.024 (-1.236, 1.285)	0.97	1.200 (0.092, 2.308)	0.034	-0.185 (-0.916, 0.546)	0.619	0.750 (0.282, 1.219)	0.002	1.301 (0.868, 1.734)	<0.001	0.436 (0.138, 0.734)	0.004
	Arm 2	0.771 (0.358, 1.183)	<0.001	0.900 (0.516, 1.285)	<0.001	0.314 (0.049, 0.579)	0.02	0.621 (0.009, 1.233)	0.047	0.822 (0.266, 1.377)	0.004	0.146 (-0.255, 0.516)	0.44	0.873 (0.314, 1.433)	0.002	0.955 (0.426, 1.483)	<0.001	0.439 (0.063, 0.815)	0.002	0.743 (-0.579, 2.065)	0.267	1.183 (0.057, 2.310)	0.04	0.281 (-0.427, 0.988)	0.435	0.773 (0.338, 1.209)	0.001	0.870 (0.459, 1.280)	<0.001	0.319 (0.033, 0.604)	0.029

	Arm 3	0.472 (0.073, 0.872)	0.02	0.468 (0.103, 0.833)	0.012	0.255 (0.004, 0.506)	0.046	0.315 (-0.191, 0.821)	0.222	0.379 (-0.073, 0.832)	0.1	0.174 (-0.130, 0.478)	0.261	0.671 (0.044, 1.297)	0.036	0.578 (0.002, 1.155)	0.049	0.363 (-0.049, 0.774))	0.084	0.637 (-0.467, 1.741)	0.255	0.567 (-0.374, 1.509)	0.234	0.324 (-0.269, 0.917)	0.283	0.448 (0.019, 0.877)	0.041	0.454 (0.058, 0.849)	0.025	0.242 (-0.034, 0.519)	0.086

CI – confidence interval, HAZ – height for age z-score, MUAC – mid-upper arm circumference, SD – standard deviation, WAZ – weight for age z-score, WHZ – weight for height z-score

Table S11. Change in child and maternal anthropometric outcomes for region and age subgroups (Incidence analysis using PP Approach)

		**Overall**						**By Region**												**By Age**											
								Bay						***Hiran***						***< 2years***						***≥ 2 years***					
**Outcome variable**	**Study Arm**	**Endline - Baseline**	**p-val**	**Midline - Baseline**	**p-val**	**Endline-Midline**	**p-val**	**Endline - Baseline**	**p-val**	**Midline - Baseline**	**p-val**	**Endline-Midline**	**p-val**	**Endline - Baseline**	**p-val**	**Midline - Baseline**	**p-val**	**Endline-Midline**	**p-val**	**Endline - Baseline**	**p-val**	**Midline - Baseline**	**p-val**	**Endline-Midline**	**p-val**	**Endline - Baseline**	**p-val**	**Midline - Baseline**	**p-val**	**Endline-Midline**	**p-val**
**Child Outcomes**																															
WHZ (mean/ 95% CI)	Arm 1	-0.017 (-0.133, 0.099)	0.776	0.080 (-0.022, 0.182)	0.126	-0.009 (-0.084, 0.067)	0.824	-0.020 (-0.213, 0.174)	0.842	0.163 (-0.002, 0.328)	0.053	-0.012 (-0.134, 0.110)	0.848	-0.015 (-0.161, 0.131)	0.836	0.039 (-0.092, 0.169)	0.561	-0.007 (-0.104, 0.090)	0.889	0.002 (-0.389, 0.393)	0.991	0.155 (-0.209, 0.518)	0.401	0.018 (-0.214, 0.250)	0.878	-0.020 (-0.140, 0.100)	0.743	0.067 (-0.035, 0.169)	0.198	-0.014 (-0.092, 0.064)	0.727
	Arm 2	0.001 (-0.110, 0.113)	0.979	0.119 (0.021, 0.216)	0.017	0.010 (-0.062, 0.083)	0.778	-0.081 (-0.253, 0.091)	0.352	0.169 (0.024, 0.314)	0.022	-0.024 (-0.133, 0.084)	0.659	0.056 (-0.092, 0.204)	0.458	0.087 (-0.043, 0.216)	0.191	0.033 (-0.065, 0.131)	0.506	0.184 (-0.176, 0.544)	0.313	0.580 (0.244, 0.916)	0.001	0.156 (-0.054, 0.367)	0.145	-0.032 (-0.148, 0.084)	0.591	0.034 (-0.063, 0.132)	0.492	-0.023 (-0.099, 0.053)	0.555
	Arm 3	-0.199 (-0.328, -0.069)	0.003	-0.062 (-0.177, 0.053)	0.288	-0.101 (-0.186, -0.016)	0.02	-0.122 (-0.291, 0.046)	0.154	0.095 (-0.049, 0.238)	0.196	-0.061 (-0.168, 0.046)	0.261	-0.293 (-0.491, -0.095)	0.004	-0.253 (-0.430, -0.075)	0.005	-0.150 (-0.283, -0.017)	0.027	-0.088 (-0.577, 0.401)	0.722	0.016 (-0.441, 0.472)	0.946	-0.063 (-0.340, 0.215)	0.656	-0.213 (-0.344, -0.082)	0.002	-0.072 (-0.185, 0.040)	0.207	-0.107 (-0.194, -0.021)	0.015
WAZ (mean/ 95% CI)	Arm 1	0.021 (-0.080, 0.121)	0.685	0.124 (0.036, 0.212)	0.006	0.017 (-0.045, 0.079)	0.593	0.070 (-0.085, 0.226)	0.375	0.175 (0.028, 0.321)	0.019	0.033 (-0.066, 0.133)	0.510	-0.004 (-0.136, 0.128)	0.948	0.098 (-0.012, 0.209)	0.080	0.009 (-0.072, 0.089)	0.831	-0.184 (-0.536, 0.167)	0.301	0.155 (-0.199, 0.509)	0.388	-0.031 (-0.252, 0.191)	0.786	0.054 (-0.048, 0.157)	0.298	0.118 (0.035, 0.202)	0.006	0.027 (-0.034, 0.087)	0.390
	Arm 2	0.085 (-0.012, 0.182)	0.085	0.191 (0.107, 0.275)	<0.001	0.050 (-0.010, 0.110)	0.104	0.053 (-0.085, 0.191)	0.453	0.252 (0.123, 0.380)	<0.001	0.045 (-0.043, 0.133)	0.315	0.106 (-0.027, 0.240)	0.118	0.152 (0.043, 0.262)	0.007	0.053 (-0.029, 0.134)	0.204	0.172 (-0.151, 0.496)	0.293	0.535 (0.207, 0.862)	0.002	0.087 (-0.114, 0.288)	0.393	0.069 (-0.030, 0.169)	0.173	0.128 (0.048, 0.208)	0.002	0.041 (-0.018, 0.100)	0.172
	Arm 3	-0.131 (-0.243, -0.019)	0.022	0.012 (-0.086, 0.111)	0.805	-0.064 (-0.133, 0.006)	0.074	-0.082 (-0.217, 0.054)	0.237	0.128 (0.001, 0.255)	0.048	-0.041 (-0.127, 0.046)	0.356	-0.191 (-0.370, -0.012)	0.036	-0.129 (-0.279, 0.021)	0.093	-0.092 (-0.202, 0.018)	0.102	-0.143 (-0.582, 0.296)	0.52	0.176 (-0.269, 0.621)	0.434	-0.033 (-0.298, 0.232)	0.809	-0.129 (-0.241, -0.017)	0.024	-0.009 (-0.101, 0.083)	0.845	-0.069 (-0.136, -0.002)	0.044
HAZ (mean/ 95% CI)	Arm 1	0.021 (-0.120, 0.161)	0.771	0.039 (-0.090, 0.169)	0.552	0.003 (-0.084, 0.091)	0.939	0.096 (-0.159, 0.350)	0.46	0.017 (-0.224, 0.258)	0.888	0.033 (-0.116, 0.182)	0.666	-0.017 (-0.184, 0.150)	0.842	0.050 (-0.100, 0.201)	0.511	-0.011 (-0.120, 0.097)	0.835	-0.464 (-0.875, -0.054)	0.027	-0.148 (-0.586, 0.290)	0.504	-0.207 (-0.468, 0.053)	0.118	0.100 (-0.048, 0.249)	0.186	0.071 (-0.063, 0.204)	0.300	0.045 (-0.046, 0.136)	0.329
	Arm 2	0.145 (0.009, 0.281)	0.037	0.177 (0.053, 0.300)	0.005	0.073 (-0.011, 0.157)	0.088	0.187 (-0.038, 0.413)	0.103	0.234 (0.022, 0.446)	0.031	0.107 (-0.025, 0.239)	0.111	0.117 (-0.053, 0.287)	0.177	0.140 (-0.010, 0.290)	0.068	0.051 (-0.058, 0.160)	0.362	-0.105 (-0.487, 0.277)	0.586	0.210 (-0.194, 0.614)	0.305	-0.131 (-0.368, 0.107)	0.280	0.190 (0.045, 0.335)	0.010	0.171 (0.043, 0.298)	0.009	0.119 (0.031, 0.207)	0.008
	Arm 3	0.016 (-0.141, 0.173)	0.846	0.060 (-0.085, 0.205)	0.419	0.013 (-0.085, 0.110)	0.802	0.060 (-0.162, 0.282)	0.592	0.070 (-0.141, 0.282)	0.514	0.027 (-0.104, 0.158)	0.685	-0.039 (-0.266, 0.187)	0.734	0.047 (-0.157, 0.252)	0.65	-0.005 (-0.152, 0.142)	0.945	-0.232 (-0.744, 0.281)	0.372	0.075 (-0.479, 0.629)	0.788	-0.005 (-0.320, 0.310)	0.975	0.047 (-0.116, 0.210)	0.573	0.058 (-0.089, 0.205)	0.440	0.015 (-0.085, 0.116)	0.763
MUAC (mean/ 95% CI)	Arm 1	0.379 (0.273, 0.485)	<0.001	0.299 (0.196, 0.402)	<0.001	0.187 (0.118, 0.257)	<0.001	0.570 (0.387, 0.753)	<0.001	0.364 (0.177, 0.551)	<0.001	0.285 (0.164, 0.406)	<0.001	0.281 (0.152, 0.410)	<0.001	0.267 (0.146, 0.388)	<0.001	0.138 (0.055, 0.222)	0.001	0.533 (0.264, 0.803)	<0.001	0.298 (0.053, 0.542)	0.018	0.218 (0.041, 0.396)	0.016	0.354 (0.238, 0.469)	<0.001	0.300 (0.187, 0.413)	<0.001	0.181 (0.106, 0.256)	<0.001
	Arm 2	0.315 (0.213, 0.417)	<0.001	0.270 (0.171, 0.368)	<0.001	0.172 (0.106, 0.239)	<0.001	0.210 (0.047, 0.373)	0.012	0.277 (0.113, 0.442)	0.001	0.102 (-0.006, 0.209)	0.063	0.384 (0.253, 0.515)	<0.001	0.265 (0.143, 0.386)	<0.001	0.218 (0.134, 0.303)	<0.001	0.359 (0.110, 0.607)	0.005	0.421 (0.195, 0.647)	<0.001	0.211 (0.049, 0.372)	0.011	0.307 (0.195, 0.419)	<0.001	0.242 (0.133, 0.350)	<0.001	0.164 (0.091, 0.237)	<0.001
	Arm 3	0.031 (-0.087, 0.150)	0.603	0.102 (-0.014, 0.217)	0.084	0.016 (-0.061, 0.093)	0.687	-0.019 (-0.179, 0.141)	0.818	-0.003 (-0.167, 0.160)	0.969	-0.009 (-0.115, 0.097)	0.863	0.093 (-0.082, 0.268)	0.298	0.230 (0.064, 0.395)	0.007	0.047 (-0.067, 0.161)	0.419	-0.084 (-0.421, 0.253)	0.622	-0.108 (-0.415, 0.199)	0.489	-0.003 (-0.216, 0.209)	0.977	0.046 (-0.081, 0.173)	0.477	0.129 (0.005, 0.254)	0.042	0.019 (-0.064, 0.102)	0.651
**Maternal Outcome**																															
Maternal MUAC (mean/	Arm 1	0.432 (-0.130, 0.994)	0.131	1.179 (0.636, 1.721)	<0.001	0.210 (-0.154, 0.574)	0.258	1.173 (0.266, 2.080)	0.012	2.362 (1.549, 3.174)	<0.001	0.521 (-0.011, 1.053)	0.055	-0.044 (-0.759, 0.671)	0.904	0.410 (-0.308, 1.129)	0.262	-0.012 (-0.513, 0.489)	0.963	-0.959 (-2.526, 0.608)	0.225	1.175 (-0.186, 2.536)	0.089	-0.539 (-1.486, 0.408)	0.262	0.597 (-0.004, 1.197)	0.052	1.179 (0.594, 1.765)	<0.001	0.317 (-0.078, 0.711)	0.116

95% CI)																															
	Arm 2	0.111 (-0.421, 0.642)	0.683	0.470 (-0.047, 0.988)	0.074	0.023 (-0.325, 0.371)	0.895	0.206 (-0.649, 1.060)	0.636	0.129 (-0.647, 0.905)	0.744	-0.103 (-0.612, 0.405)	0.69	0.049 (-0.630, 0.727)	0.888	0.691 (0.007, 1.375)	0.048	0.114 (-0.365, 0.593)	0.641	0.247 (-1.235, 1.730)	0.739	0.942 (-0.307, 2.191)	0.136	0.104 (-0.754, 0.962)	0.811	0.094 (-0.474, 0.663)	0.744	0.413 (-0.147, 0.974)	0.148	0.010 (-0.370, 0.391)	0.958
	Arm 3	0.243 (-0.336, 0.821)	0.41	0.280 (-0.280, 0.840)	0.327	0.149 (-0.226, 0.524)	0.436	0.045 (-0.737, 0.826)	0.91	0.351 (-0.353, 1.055)	0.326	0.070 (-0.394, 0.534)	0.767	0.494 (-0.371, 1.359)	0.262	0.189 (-0.682, 1.060)	0.669	0.258 (-0.350, 0.867)	0.404	1.289 (-0.234, 2.812)	0.095	1.311 (0.028, 2.595)	0.045	0.480 (-0.387, 1.346)	0.276	0.102 (-0.521, 0.725)	0.747	0.139 (-0.472, 0.751)	0.654	0.087 (-0.327, 0.501)	0.68

CI – confidence interval, HAZ – height for age z-score, MUAC – mid-upper arm circumference, SD – standard deviation, WAZ – weight for age z-score, WHZ – weight for height z-score

Figure S2(a). Child wasting outcome by WHZ using different analysis approaches

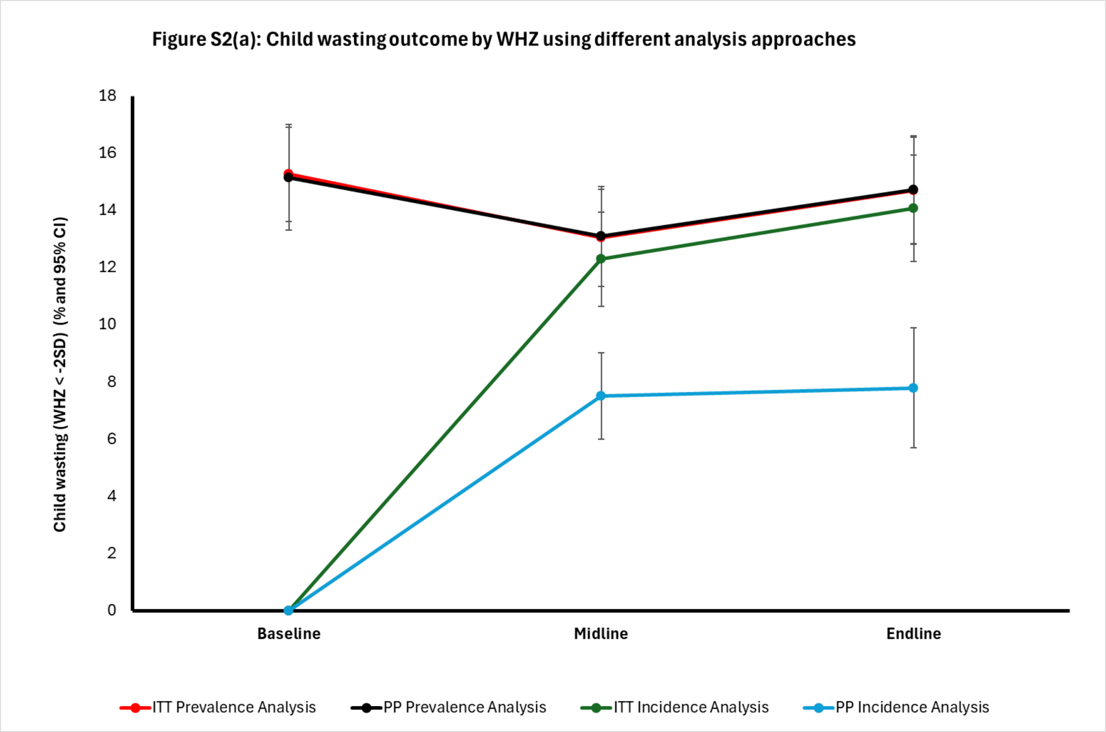

Figure S2(b). Maternal wasting outcome by MUAC using different analysis approaches

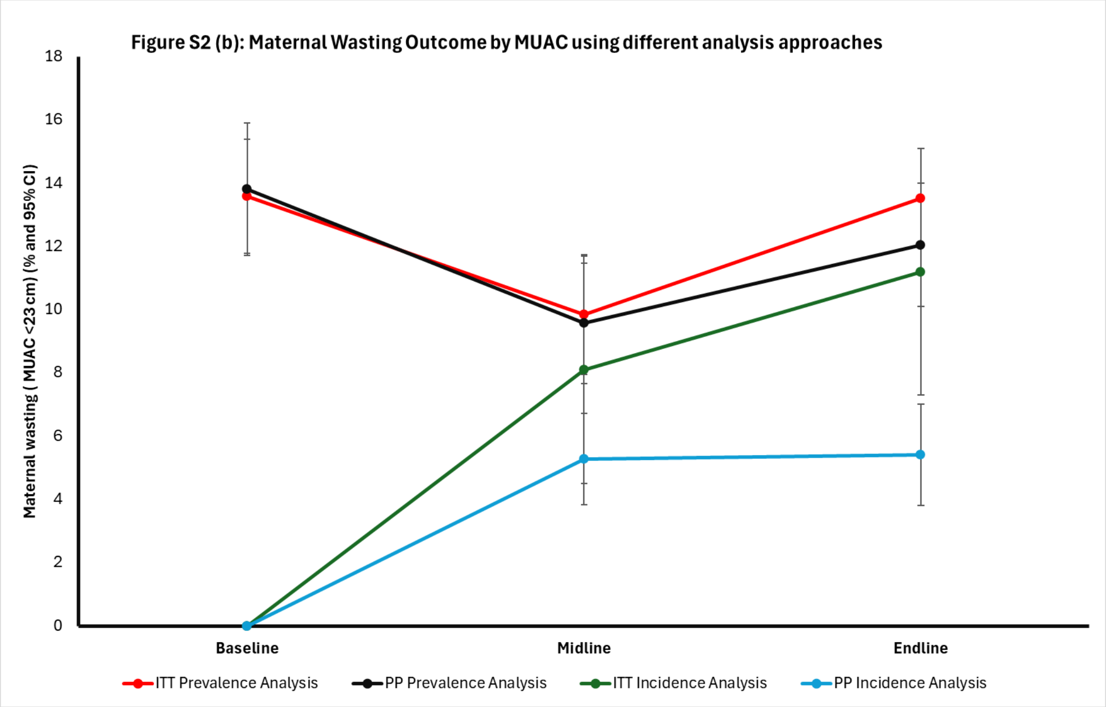

Table S12. Change in child and maternal anthropometric outcomes: WAZ, HAZ, Maternal MUAC (Prevalence analysis using ITT Approach)

		**Unadjusted**						**Partially Adjusted**						**Fully Adjusted**					
**Outcome variable**	**Study Arm**	**Endline - Baseline**	**p-val**	**Midline - Baseline**	**p-val**	**Endline-Midline**	**p-val**	**Endline - Baseline**	**p-val**	**Midline - Baseline**	**p-val**	**Endline-Midline**	**p-val**	**Endline - Baseline**	**p-val**	**Midline - Baseline**	**p-val**	**Endline-Midline**	**p-val**
**Child Outcomes**																			
WAZ (mean/ 95% CI)	Arm 1	**0.169 (0.082, 0.257)**	**<0.001**	**0.221 (0.144, 0.298)**	**<0.001**	**0.099 (0.048, 0.151)**	**<0.001**	**0.302 (0.107, 0.496)**	**0.002**	**0.466 (0.300, 0.632)**	**<0.001**	**0.220 (0.107, 0.332)**	**<0.001**	-0.064 (-0.307, 0.178)	0.603	**0.330 (0.118, 0.543)**	**0.002**	0.048 (-0.097, 0.193)	0.517
	Arm 2	**0.233 (0.148, 0.319)**	**<0.001**	**0.310 (0.235, 0.384)**	**<0.001**	**0.122 (0.071, 0.173)**	**<0.001**	**0.433 (0.174, 0.692)**	**0.001**	**0.593 (0.373, 0.813)**	**<0.001**	**0.271 (0.121, 0.420)**	**<0.001**	0.120 (-0.182, 0.422)	0.435	**0.505 (0.241, 0.768)**	**<0.001**	0.123 (-0.058, 0.303)	0.183
	Arm 3	0.074 (-0.015, 0.163)	0.103	**0.143 (0.066, 0.221)**	**<0.001**	0.051 (-0.003, 0.104)	0.063	0.343 (-0.037, 0.723)	0.076	**0.456 (0.132, 0.780)**	**0.006**	**0.228 (0.006, 0.450)**	**0.044**	0.068 (-0.342, 0.478)	0.745	**0.405 (0.047, 0.762)**	**0.027**	0.092 (-0.154, 0.339)	0.463
HAZ (mean/ 95% CI)	Arm 1	0.120 (-0.004, 0.244)	0.059	**0.135 (0.024, 0.246)**	**0.017**	0.062 (-0.010, 0.134)	0.094	0.172 (-0.105, 0.449)	0.225	0.197 (-0.047, 0.441)	0.113	0.098 (-0.060, 0.255)	0.223	-0.004 (-0.348, 0.341)	0.983	0.164 (-0.146, 0.475)	0.299	-0.002 (-0.205, 0.201)	0.986
	Arm 2	**0.250 (0.129, 0.371)**	**<0.001**	**0.241 (0.134, 0.348)**	**<0.001**	**0.121 (0.050, 0.191)**	**0.001**	0.302 (-0.066, 0.671)	0.108	0.276 (-0.048, 0.599)	0.095	0.136 (-0.073, 0.346)	0.202	0.114 (-0.315, 0.544)	0.602	0.245 (-0.139, 0.629)	0.210	0.034 (-0.219, 0.286)	0.792
	Arm 3	**0.185 (0.059, 0.312)**	**0.004**	**0.120 (0.008, 0.233)**	**0.036**	**0.088 (0.013, 0.162)**	**0.021**	0.200 (-0.341, 0.741)	0.469	0.087 (-0.389, 0.562)	0.721	0.053 (-0.257, 0.364)	0.736	-0.113 (-0.696, 0.470)	0.703	-0.036 (-0.557, 0.484)	0.891	-0.114 (-0.458, 0.231)	0.517
**Maternal Outcome**																			
Maternal MUAC, cm (mean/ 95% CI)	Arm 1	**0.665 (0.227, 1.104)**	**0.003**	**1.290 (0.887, 1.693)**	**<0.001**	**0.367 (0.101, 0.633)**	**0.007**	-0.219 (-1.225, 0.787)	0.669	**1.283 (0.348, 2.217)**	**0.007**	0.123 (-0.474, 0.720)	0.686	-0.208 (-1.441, 1.026)	0.741	0.971 (-0.171, 2.112)	0.095	0.125 (-0.622, 0.872)	0.743
	Arm 2	**0.771 (0.358, 1.183)**	**<0.001**	**0.900 (0.516, 1.285)**	**<0.001**	**0.335 (0.076, 0.593)**	**0.011**	-0.627 (-1.926, 0.672)	0.343	0.751 (-0.451, 1.953)	0.220	0.004 (-0.771, 0.779)	0.992	-0.658 (-2.167, 0.850)	0.392	0.352 (-1.032, 1.736)	0.618	-0.016 (-0.927, 0.895)	0.972
	Arm 3	**0.472 (0.073, 0.872)**	**0.020**	**0.468 (0.103, 0.833)**	**0.012**	0.223 (-0.024, 0.469)	0.076	-1.653 (-3.569, 0.263)	0.091	0.163 (-1.604, 1.930)	0.856	-0.222 (-1.365, 0.921)	0.703	-1.720 (-3.802, 0.361)	0.105	-0.210 (-2.122, 1.703)	0.830	-0.257 (-1.510, 0.996)	0.688

CI – confidence interval, CM – centimeters, HAZ – height for age z-score, MUAC – mid-upper arm circumference, WAZ – weight for age z-score

*Footnote 1. Partially adjusted for cluster, child age and sex.
†Footnote 2. Fully adjusted for cluster, child age, sex, displacement status, wasting , underweight, stunting at baseline, maternal education HH monthly expenditure at baseline, sanitation and access to protected water source at baseline

Table 13. Crude and adjusted logistic regression showing difference-in-differences effects for child anthropometry outcomes (Prevalence analysis using ITT Approach)

**Outcome variable**		**Unadjusted model**				**Partially Adjusted model §**				**Fully Adjusted model ¶**			
	**Study Arm**	**Midline**	**p-val**	**Endline**	**p-val**	**Midline**	**p-val**	**Endline**	**p-val**	**Midline**	**p-val**	**Endline**	**p-val**
**Child Outcomes**		**(*n=1,607)**		**(n=1,473)**		**(n=1,607)**		**(n=1,473)**		**(n=1,607)**		**(n=1,473)**	
WHZ < -2 SD (†OR, 95% CI)	Arm 2	**0.57 (0.312, 1.034)**	**0.064**	**0.53 (0.289, 0.972)**	**0.040**	**0.53 (0.290, 0.980)**	**0.043**	**0.52 (0.279, 0.956)**	**0.035**	**0.55 (0.302, 1.017)**	**0.057**	**0.52 (0.283, 0.967)**	**0.039**
	Arm 3	1.21 (0.671, 2.183)	0.525	1.24 (0.687, 2.249)	0.472	1.21 (0.667, 2.210)	0.526	1.31 (0.720, 2.383)	0.376	1.24 (0.677, 2.284)	0.483	1.34 (0.730, 2.460)	0.346
WAZ < -2 SD (OR, 95% CI)	Arm 2	1.09 (0.550, 2.166)	0.803	0.68 (0.334, 1.367)	0.276	1.08 (0.543, 2.163)	0.819	0.66 (0.325, 1.340)	0.250	1.04 (0.510, 2.135)	0.907	0.61 (0.293, 1.267)	0.185
	Arm 3	1.35 (0.702, 2.585)	0.370	1.62 (0.848, 3.099)	0.144	1.36 (0.706, 2.620)	0.359	1.62 (0.843, 3.107)	0.148	1.24 (0.629, 2.447)	0.535	1.54 (0.788, 3.021)	0.206
HAZ < -2 SD (OR, 95% CI)	Arm 2	1.23 (0.645, 2.330)	0.534	0.83 (0.431, 1.600)	0.578	1.17 (0.615, 2.226)	0.632	0.78 (0.405, 1.507)	0.461	1.14 (0.586, 2.199)	0.707	0.75 (0.382, 1.468)	0.400
	Arm 3	1.39 (0.754, 2.578)	0.290	1.10 (0.588, 2.050)	0.769	1.35 (0.730, 2.495)	0.340	1.05 (0.564, 1.966)	0.870	1.19 (0.629, 2.247)	0.593	0.95 (0.499, 1.808)	0.876
MUAC < 11.5cm (OR, 95% CI)	Arm 2	1.59 (0.260, 9.730)	0.616	N/A ‡	-	1.63 (0.268, 9.912)	0.595	N/A ‡	-	1.72 (0.276, 10.740)	0.561	N/A ‡	-
	Arm 3	2.29 (0.210, 25.030)	0.497	1.86 (0.184, 18.781)	0.598	2.22 (0.205, 24.111)	0.512	1.79 (0.178, 17.929)	0.621	2.41 (0.214, 27.016)	0.477	1.92 (0.184, 20.057)	0.585

CI – confidence interval, CM – centimetres, HAZ – height for age z-score, ITT – intention to treat, MUAC – mid-upper arm circumference, OR – odds ratio, SD – standard deviation, WAZ – weight for age z-score, WHZ – weight for height z-score

*Footnote 1. n for child outcome is number of U5 children analyzed
†Footnote 2. Arm 1 is the reference arm for the odds ratio
‡Footnote 3. N/A: No data point, omitted due to collinearity
§Footnote 4. Partially adjusted for cluster, child age, sex and wasting at baseline
¶Footnote 5. Fully adjusted for cluster, child age, sex, displacement status, wasting, underweight, stunting at baseline, maternal education HH monthly expenditure at baseline, sanitation and access to protected water source at baseline

Table S14. Unadjusted difference in differences of child and maternal wasting outcomes (Prevalence analysis using ITT Approach)

		**Region**								**Age**							
		**Bay**				**Hiran**				**< 2 years**				**≥ 2 years**			
**Outcome variable**	**Study Arm**	**Midline**	**p-val**	**Endline**	**p-val**	**Midline**	**p-val**	**Endline**	**p-val**	**Midline**	**p-val**	**Endline**	**p-val**	**Midline**	**p-val**	**Endline**	**p-val**
**Child Outcomes**																	
WHZ < -2 SD (OR, 95% CI)	Arm 2	0.49 (0.125, 1.881)	0.295	0.58 (0.178, 1.914)	0.374	0.62 (0.320, 1.204)	0.158	0.54 (0.271, 1.079)	**0.081**	0.60 (0.128, 2.768)	0.509	0.60 (0.109, 3.307)	0.558	0.55 (0.288, 1.069)	0.078	0.51 (0.265, 0.992)	**0.047**
	Arm 3	1.56 (0.427, 5.710)	0.501	1.48 (0.451, 4.875)	0.517	1.20 (0.615, 2.346)	0.592	1.27 (0.639, 2.525)	0.495	1.78 (0.394, 8.043)	0.454	2.80 (0.548, 14.346)	0.216	1.11 (0.583, 2.114)	0.751	1.07 (0.562, 2.041)	0.835
WAZ < -2 SD (OR, 95% CI)	Arm 2	0.47 (0.158, 1.385)	0.170	0.26 (0.087, 0.795)	**0.018**	1.95 (0.793, 4.805)	0.145	1.32 (0.519, 3.340)	0.563	0.30 (0.065, 1.418)	0.130	0.49 (0.100, 2.407)	0.380	1.58 (0.716, 3.507)	0.256	0.80 (0.358, 1.807)	0.597
	Arm 3	0.84 (0.325, 2.175)	0.721	0.93 (0.366, 2.374)	0.884	1.87 (0.737, 4.725)	0.188	2.40 (0.941, 6.116)	**0.067**	0.91 (0.264, 3.145)	0.884	0.61 (0.147, 2.502)	0.489	1.57 (0.720, 3.435)	0.256	2.24 (1.042, 4.796)	**0.039**
HAZ < -2 SD (OR, 95% CI)	Arm 2	1.00 (0.395, 2.539)	0.998	0.51 (0.199, 1.313)	0.163	1.36 (0.558, 3.335)	0.495	1.20 (0.476, 3.040)	0.695	0.73 (0.237, 2.270)	0.590	0.24 (0.064, 0.908)	**0.035**	1.56 (0.702, 3.461)	0.275	1.36 (0.609, 3.056)	0.450
	Arm 3	1.19 (0.510, 2.765)	0.691	0.73 (0.312, 1.721)	0.475	1.27 (0.501, 3.196)	0.618	1.29 (0.497, 3.371)	0.598	1.19 (0.407, 3.479)	0.751	0.63 (0.184, 2.146)	0.458	1.43 (0.665, 3.087)	0.359	1.34 (0.618, 2.916)	0.456
MUAC < 11.5cm (OR, 95% CI)	Arm 2	0.05 (0.001, 2.616)	0.141	N/A	-	2.14 (0.292, 15.644)	0.455	N/A	-	0.23 (0.010, 5.489)	0.367	N/A	-	4.51 (0.343, 59.436)	0.252	N/A	-
	Arm 3	N/A	-	N/A	-	2.01 (0.121, 33.390)	0.626	3.36 (0.226, 49.767)	0.379	10.78 (0.485, 239.823)	0.133	11.76 (0.342, 404.668)	0.172	0.31 (0.019, 5.310)	0.423	N/A	-
**Maternal Outcome**																	
MUAC < 23cm (OR, 95% CI)	Arm 2	2.51 (0.603, 10.436)	0.206	1.37 (0.384, 4.888)	0.628	2.13 (0.746, 6.079)	0.158	0.66 (0.249, 1.725)	0.392	1.93 (0.229, 16.36)	0.544	0.90 (0.107, 7.589)	0.924	2.51 (0.992, 6.374)	0.052	0.89 (0.0.384, 2.055)	0.783
	Arm 3	2.87 (0.813, 10.158)	0.101	1.207 (0.385, 3.788)	0.747	0.87 (0.287, 2.620)	0.801	0.54 (0.199, 1.438)	0.215	0.76 (0.130, 4.439)	0.760	0.35 (0.058, 2.091)	0.249	1.74 (0.688, 4.401)	0.242	0.81 (0.353, 1.860)	0.620

CI – confidence interval, CM – centimeters, HAZ – height for age z-score, IDP – internally displaced person, MUAC – mid-upper arm circumference, OR – odds ratio SD – standard deviation, WAZ – weight for age z-score, WHZ – weight for height z-score

*Footnote 1. Arm 1 is the reference arm
†Footnote 2. N/A: No data point
‡Footnote 3. Partially adjusted for cluster, child age, sex and wasting at baseline.
§Footnote 4. Fully adjusted for cluster, child age, sex, displacement status, wasting , underweight, stunting at baseline, maternal education HH monthly expenditure at baseline, sanitation and access to protected water source at baseline

Figure S3. Maternal wasting prevalence using MUAC by Arm (ITT analysis)

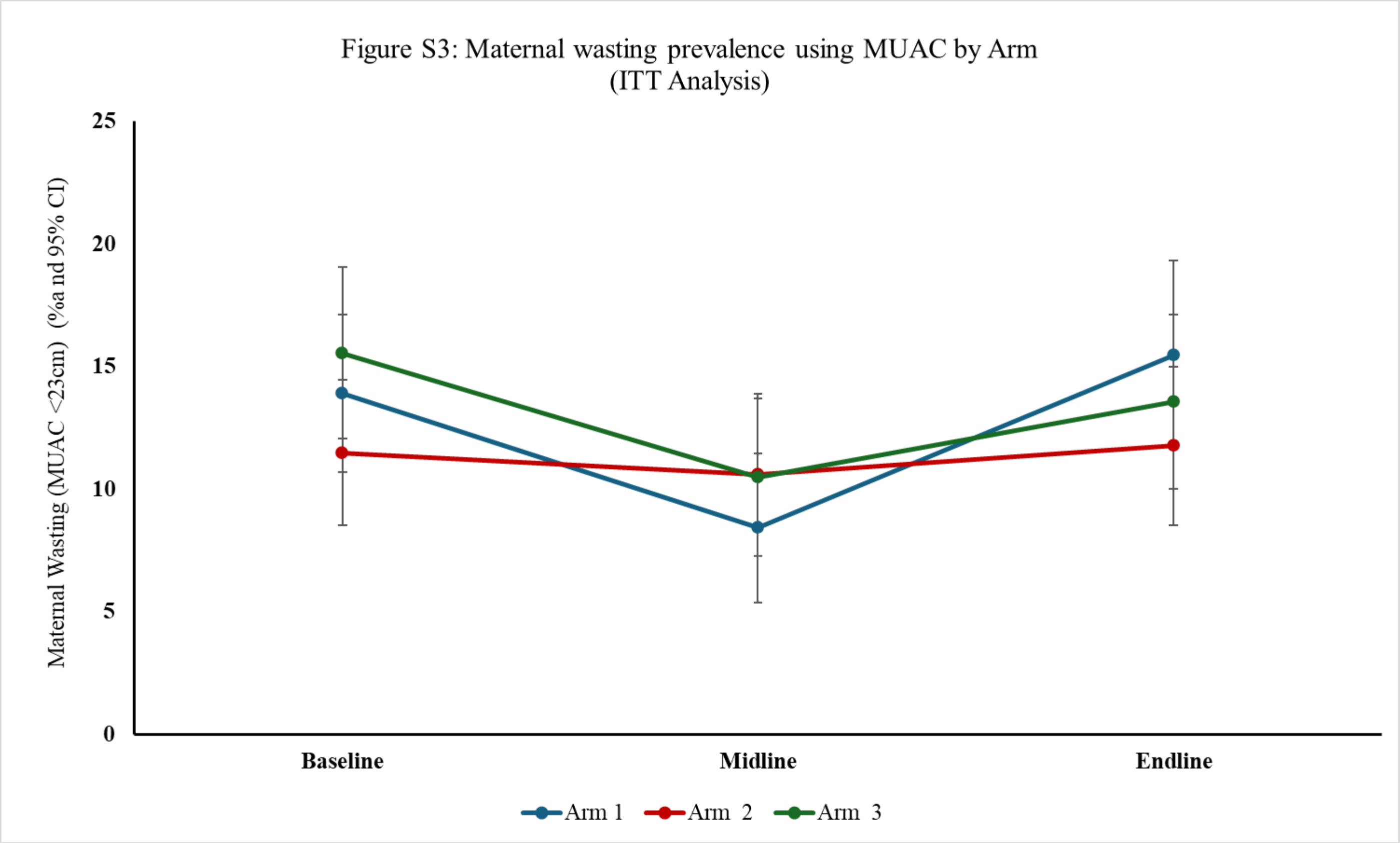

Table S15: Unadjusted and adjusted difference in differences of child and maternal wasting outcomes by region (Prevalence analysis using ITT Approach) †

		Unadjusted								Adjusted ‡							
		Bay (IDP) §				Hiran (Host community) §				Bay (IDP)				Hiran (Host community)			
Outcome variable	Study Arm	Midline	p-val	Endline	p-val	Midline	p-val	Endline	p-val	Midline	p-val	Endline	p-val	Midline	p-val	Endline	p-val
Child Outcomes																	
WHZ < -2 SD (†OR, 95% CI)	Arm 2	0.49 (0.125, 1.881)	0.295	0.58 (0.178, 1.914)	0.374	0.62 (0.320, 1.204)	0.158	0.54 (0.271, 1.079)	0.081	0.47 (0.12, 1.90)	0.29	0.55 (0.15, 1.95)	0.352	0.60 (0.31, 1.18)	0.137	0.52 (0.26, 1.06)	0.071
	Arm 3	1.56 (0.427, 5.710)	0.501	1.48 (0.451, 4.875)	0.517	1.20 (0.615, 2.346)	0.592	1.27 (0.639, 2.525)	0.495	1.98 (0.51, 7.68)	0.325	1.83 (0.52, 6.41)	0.347	1.15 (0.58, 2.27)	0.691	1.26 (0.63, 2.52)	0.518
WAZ < -2 SD (OR, 95% CI)	Arm 2	0.47 (0.158, 1.385)	0.170	0.26 (0.087, 0.795)	0.018	1.95 (0.793, 4.805)	0.145	1.32 (0.519, 3.340)	0.563	0.38 (0.12, 1.19)	0.096	0.20 (0.06, 0.63)	0.006	2.10 (0.82, 5.36)	0.121	1.40 (0.53, 3.68)	0.494
	Arm 3	0.84 (0.325, 2.175)	0.721	0.93 (0.366, 2.374)	0.884	1.87 (0.737, 4.725)	0.188	2.40 (0.941, 6.116)	0.067	0.74 (0.27, 1.98)	0.546	0.81 (0.31, 2.13)	0.675	1.77 (0.67, 4.65)	0.249	2.52 (0.96, 6.67)	0.062
HAZ < -2 SD (OR, 95% CI)	Arm 2	1.00 (0.395, 2.539)	0.998	0.51 (0.199, 1.313)	0.163	1.36 (0.558, 3.335)	0.495	1.20 (0.476, 3.040)	0.695	0.93 (0.36, 2.40)	0.88	0.46 (0.18, 1.20)	0.111	1.39 (0.55, 3.53)	0.49	1.28 (0.49, 3.35)	0.614
	Arm 3	1.19 (0.510, 2.765)	0.691	0.73 (0.312, 1.721)	0.475	1.27 (0.501, 3.196)	0.618	1.29 (0.497, 3.371)	0.598	1.07 (0.45, 2.53)	0.879	0.65 (0.27, 1.54)	0.325	1.10 (0.41, 2.92)	0.852	1.27 (0.46, 3.46)	0.647
MUAC < 11.5cm (OR, 95% CI)	Arm 2	0.05 (0.001, 2.616)	0.141	N/A*	-	2.14 (0.292, 15.644)	0.455	N/A*	-	0.11 (0.01, 2.22)	0.151	N/A*	-	2.19 (0.30, 16.13)	0.443	-	-
	Arm 3	N/A*	-	N/A*	-	2.01 (0.121, 33.390)	0.626	3.36 (0.226, 49.767)	0.379	N/A	-	N/A*	-	1.99 (0.12, 33.50)	0.632	3.61 (0.24, 54.64)	0.355
Maternal Outcome																	
MUAC < 23cm (OR, 95% CI)	Arm 2	2.51 (0.603, 10.436)	0.206	1.37 (0.384, 4.888)	0.628	2.13 (0.746, 6.079)	0.158	0.66 (0.249, 1.725)	0.392	1.73 (0.43, 7.00)	0.439	1.22 (0.35, 4.19)	0.753	1.63 (0.62, 4.31)	0.325	0.55 (0.21, 1.46)	0.232
	Arm 3	2.87 (0.813, 10.158)	0.101	1.207 (0.385, 3.788)	0.747	0.87 (0.287, 2.620)	0.801	0.54 (0.199, 1.438)	0.215	2.88 (0.83, 9.96)	0.095	1.13 (0.37, 3.47)	0.837	0.84 (0.30, 2.35)	0.737	0.57 (0.21, 1.53)	0.265

CI – confidence interval, CM – centimeters, HAZ – height for age z-score, IDP – internally displaced person, ITT – intention to treat, MUAC – mid-upper arm circumference, OR – odds ratio, SD – standard deviation, WAZ – weight for age z-score, WHZ – weight for height z-score

*Footnote 1. N/A: No data point

†Footnote 2. Arm 1 is the reference arm for the odds ratio

‡Footnote 3. Adjusted for child age, sex, wasting , underweight, stunting at baseline, maternal education HH monthly expenditure at baseline, sanitation and access to protected water source at baseline

§Footnote 4. Bay region was IDPs and Hiran host community

Table S16. Cost Analysis Indicators Compared Across Study Arms

	**Arm 1**	**Arm 2**	**Arm 3**
	**(cash only)**	**(cash + SBCC)**	**(cash + top-up)**
**Cost Efficiency**			
Average cost per beneficiary household (excluding societal costs)	$875	$906	$1,204
Total cost per beneficiary household (including societal costs)	$876	$914	$1,205
Cash Transfer Ratio, gross transfer	$0.80	$0.80	$0.69
**Cost-Effectiveness**			
(wasting defined using WHZ <-2)			
% Reduction in wasting prevalence (from baseline to endline)	-0.30%	4.90%	-1.90%
**Societal Costs**			
Societal costs per beneficiary household	$1	$8	$1
Societal cost to beneficiaries, as a proportion of total cost per beneficiary household	0.06%	0.91%	0.05%

Figure S4. Qualitative results

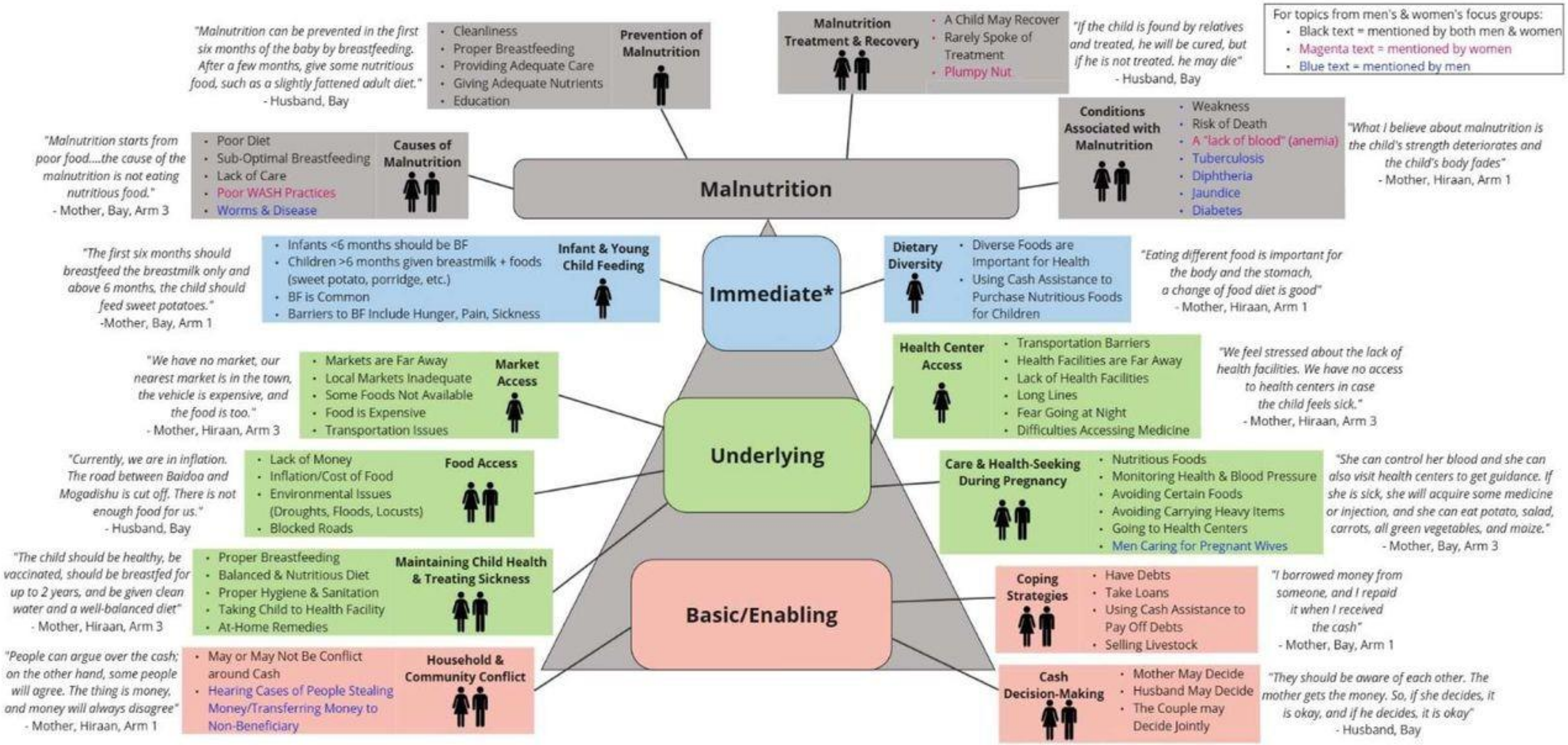

In the focus groups, women were asked questions around infant and child diet and feeding, breastfeeding practices and challenges, household food consumption, food and market access, dietary diversity, maintaining child health and caring for sick children, views of wasting, care and health-seeking practices during pregnancy, coping strategies for debt, decision-making over cash, and household and/or community conflict related to cash. Similar topics were explored in the men's focus groups, however, some questions were adapted to capture the man's perspective and role. Additionally, men were also asked about the prevention of malnutrition. The icons represent whether a topic was explored/discussed in the men's and/or women's focus groups. *Additional immediate-level topics are presented in the corresponding table.

Figure S5. Household Expenditures at Baseline, Midline, and Endline

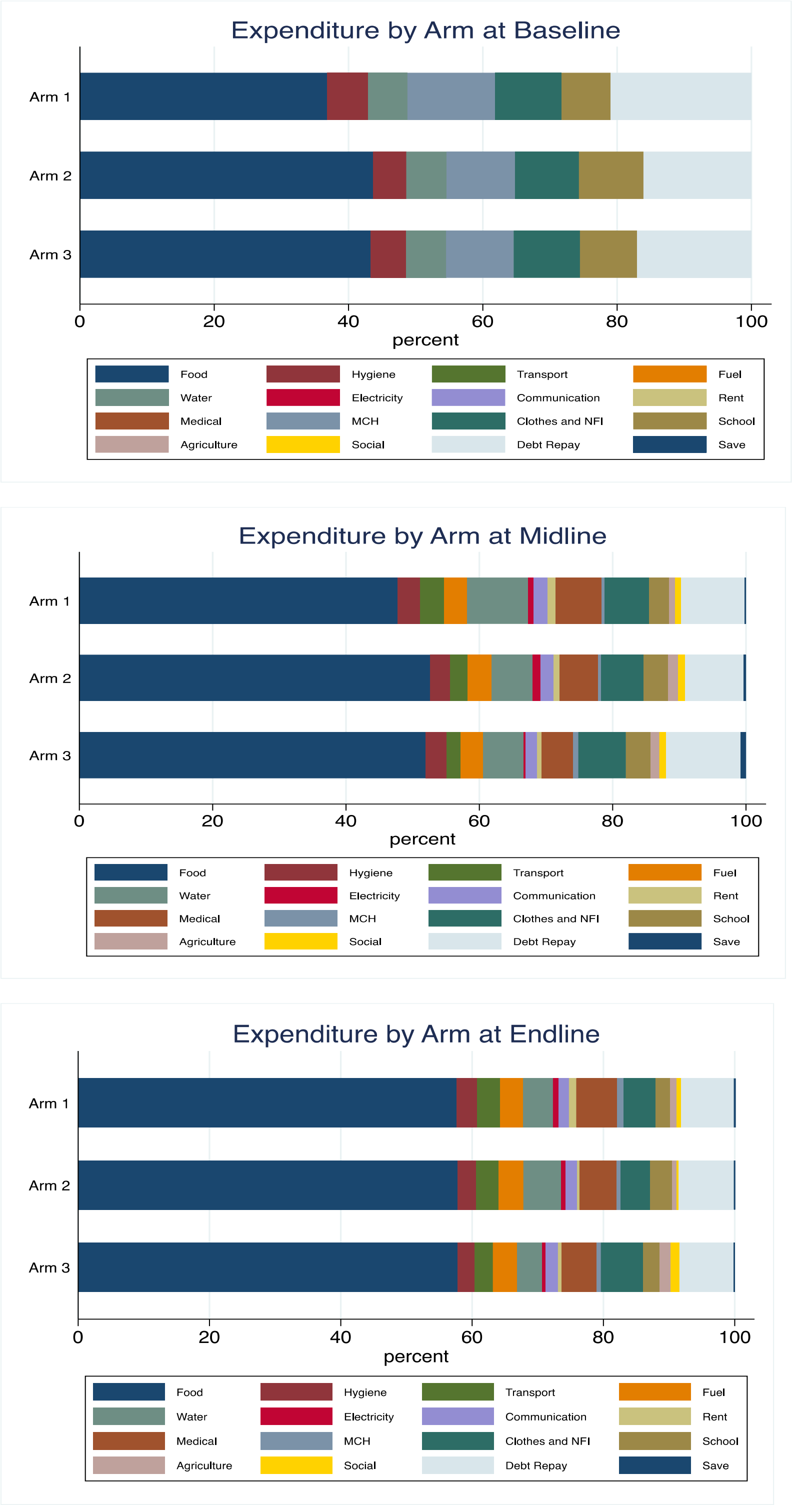

Text S6. Effectiveness and Cost-Effectiveness of Cash-Based Interventions on Nutrition Outcomes

Cash-based interventions (CBIs) have been shown to be more effective at decreasing food insecurity and malnutrition, especially in low- and middle-income countries and humanitarian contexts.[1] CBIs can vary, ranging from straightforward unconditional cash transfers to more established programs like conditional transfers or voucher programs. CBIs aim to enhance access to nutritious foods and general nutrition. CBIs may also be employed to achieve broader health outcomes related to maternal and child health, such as wasting and stunting.

This literature review discusses the impacts of CBIs on humanitarian and low-income nutritional outcomes in the form of malnutrition reduction, dietary diversity, and cost-effectiveness. Therefore, a targeted review of five studies and one report was employed to examine conditional and unconditional cash transfers in environments with high food insecurity. Studies were chosen based on their nutritional effect assessments and relevance to CBIs conducted in humanitarian or low-income settings.

The studies examined programs in Pakistan, Syria, Niger, and Burkina Faso with various CBI designs. In Niger, one study compared the costs, cost-effectiveness, and cost-efficiency of two cash-based interventions for preventing acute malnutrition in children under five years. It compared a modified cash transfer program, which began earlier and ran longer to a standard program.[2] In Pakistan, a trial evaluated the impact of three CBIs (standard cash, double cash, and fresh food vouchers) on child nutrition outcomes in a humanitarian context. It considered the effects on stunting and wasting at six months and one year and on enhancing nutritional resilience.[1,3] In Burkina Faso, research was concerned with the cost and cost-effectiveness of mobile cash transfers for the MAM'Out project, adopting a societal perspective as it included costs falling on implementing partners, as well as beneficiary households.[4] A study in Northern Syria examined the feasibility of CBIs, their acceptability, infrastructure, capacity for implementation, value for money, risks, and responsiveness to the needs of beneficiaries. The study aimed to guide the way forward in humanitarian aid approaches and steer the scale-up of cash-based modality in a context historically dominated by in-kind programming.[5]

This review highlights key parameters of intervention effectiveness, including program design and education components, as well as external factors such as conflict and displacement.

Overall Effectiveness of CBIs on Nutrition Outcomes

Across several studies, there is a growing consensus that cash transfers can improve nutrition outcomes, particularly for children and mothers. However, their effectiveness varies based on factors such as transfer modality, conditionality, program duration, and complementary support services. Studies from Sindh Province, Pakistan, and Tapoa Province, Burkina Faso, both humanitarian settings where CBIs were used to address food insecurity. In Pakistan, a trial assessed the impact of unconditional cash transfers: Standard Cash (SC), Double Cash (DC), alongside Fresh Food Vouchers (FFV) on child nutrition.[1,3] In Burkina Faso, research focused on the cost-effectiveness of mobile unconditional cash transfers under the MAM'Out project, with community sensitization sessions encouraging spending on food.[4] By comparing these approaches, this review explores how cash transfer modalities and program structures influence nutrition outcomes in humanitarian settings.

One key finding from successful CBI programs is their impact on dietary diversity. Unconditional cash transfers often enable households to improve their food purchasing, leading to higher-quality diets. This is particularly important for households facing extreme poverty or seasonal food shortages.[4] For instance, a study conducted in Pakistan, a cluster-randomized controlled trial evaluating different CBIs, assessed the effects of SC, DC, FFV on maternal and child nutrition outcomes.[1] While it was initially hypothesized that FFVs would have the greatest impact on child growth and micronutrient status by increasing dietary diversity, the results showed a more significant improvement in dietary diversity for the DC arm, followed by SC, with FFV showing the least

improvement. However, in terms of weight-for-height z-scores (WHZ), children in the FFV arm experienced greater growth compared to those in the SC arm.[1]

According to evidence from child nutrition studies, cash transfers can help reduce stunting and wasting in children under the age of five, especially when accompanied by nutrition instruction or counseling[4]. In Burkina Faso, the MAM'Out initiative found that conditional cash transfers combined with nutrition instruction had a considerably higher impact on child nutrition outcomes than programs that offered neither cash nor educational support[4]. This emphasizes the need of combining financial support with SBCC measures to increase the effectiveness of CBIs in combating malnutrition.[4]

CBI success is affected by a range of programmatic and environmental factors. Cash transfer amounts, intervention duration, and whether the program has complementary services (e.g., health access, nutrition counseling) all impact results. Modality of transfer and delivery mechanisms are also crucial. Mobile cash transfer mechanisms, for example, can be used to boost access in areas with fewer banks, while direct cash transfers may prove more effective in settings where digital payment infrastructure is low.[1,4] It is therefore important to consider these context-specific factors to optimize CBIs and ensure that they become effective in improving food security and reducing malnutrition among the target groups.

Cost-Effectiveness of CBIs

Understanding the cost-effectiveness of CBI's is crucial, particularly when compared to in-kind food aid. This review examines cost-effectiveness by analyzing peer-reviewed studies from Burkina Faso, Syria, and Pakistan, along with an organizational report from the Cash Alliance on emergency cash support in Somalia. The Burkina Faso study assessed the cost-efficiency of mobile unconditional cash transfers, while research in Syria examined the feasibility and value for money of CBIs in a conflict-affected region.[4,5] A Pakistan study evaluated the cost-effectiveness of SC, DC, and FFV in improving child nutrition outcomes.[3] Findings suggest that while CBIs can be a cost-effective alternative to in-kind aid, efficiency varies based on delivery mechanisms and impact per dollar spent.

CBIs are often more cost-efficient than in-kind food assistance due to lower logistical and operational costs.[5] Unlike food aid, cash does not require extensive infrastructure for storage, transportation, or distribution, making it a more scalable option, particularly in humanitarian settings where resources are limited.[5] Mobile transfers further enhance efficiency by reducing costs associated with manual cash distribution while ensuring direct payments to recipients, minimizing intermediary handling and corruption risks.[5] For instance, mobile cash transfers in Somalia allowed beneficiaries to receive funds even if they relocated, making them particularly beneficial for internally displaced persons.[6] While cash transfers were generally more cost-effective, the specific delivery mechanism had an impact on efficiency, with mobile transfers proving to be a cost-saving option to traditional distribution techniques. However, one study also revealed that cost-efficiency is not universal across all CBIs. A study in Dadu, Pakistan, which evaluated the cost-efficiency of three CBI models: SC, DC, and FFV, found that cash beneficiaries incurred significantly higher personal costs compared to voucher recipients.[3] Unlike voucher distribution, which took place directly in beneficiaries' villages, most cash recipients had to travel to collect their payments, sometimes spending an entire day due to public transportation schedules.[3] This suggests that while CBIs can reduce overall program costs, their design must consider beneficiary accessibility to avoid unintended financial burdens.

Several studies have found that CBIs, particularly unconditional cash transfers, provide better returns on investment in terms of nutrition intake and improvement.[1,3,4] The flexibility of cash allows recipients to allocate funds according to their specific needs, maximizing the effectiveness of each dollar spent. In contrast, food aid and voucher systems impose spending restrictions, which can limit their overall cost-effectiveness by preventing households from prioritizing essential nutritional needs.[5]

However, financial flexibility alone does not guarantee improved dietary outcomes. While CBIs allow households to allocate resources according to their needs, their impact depends on market access and recipient knowledge. If beneficiaries lack nutrition education or access to affordable and diverse food options, the benefits of cash transfers may be limited. Globally, the way markets function in crises remains poorly understood, and the comparative impact of different humanitarian assistance modalities on market dynamics is still an area of ongoing research.[5] Market disruptions, inflation, or supply shortages can reduce the purchasing power of cash assistance, making it less effective than in-kind food aid in certain contexts.[5] Therefore, conducting market assessments in target areas is essential to determine the feasibility of CBIs as a substitute for traditional food assistance.

Specific Interventions and their Impacts

The studies show that the design of cash interventions has a major impact on their effectiveness. Some of the intervention models include:

Unconditional Cash transfers (UCTs)

UCTs provide recipients with financial support without strings attached, and the freedom to use it as per immediate needs. They are greatly appreciated in humanitarian settings for the timeliness of their rollout and for the dignity of allowing recipients to make their own financial decisions. However, while UCTs have been shown to improve food security, they may not always lead to significant health and nutrition change in children in the absence of complementary support.[3] Some limiting factors include beneficiaries' low nutritional knowledge and households' limited market access to a diversity of affordable foods, which can prevent households from making the most nutritionally effective purchases. A study in Somalia found that the combination of nutrition counseling with UCTs was more effective in improving the growth of children and household food security than UCTs alone.[7] This emphasizes the requirement to combine UCTs with complementary interventions, such as nutrition education and health.

Conditional Cash Transfers (CCTs)

CCTs only pay when the beneficiary meets specified conditions, such as going to health centers, participating in nutrition education classes, or enrolling in community-based behavior programs. By linking cash assistance to health and education activities, CCTs can lead to significant longer-term changes in child health, care consumption, and well-being compared to unconditional transfers.[5] These programs promote health-seeking behaviors, and the beneficiaries are able to receive the required services that can enhance their nutritional status.[5] While CCTs have demonstrated stronger impacts on long-term health and education, their success depends on program design, implementation, and access to required services. In settings where health facilities or nutrition programs are not readily available, it may be hard to achieve CCT requirements, limiting the reach and impact of these interventions.[5]

Double Cash (DC)

DC transfers involve providing double the value of cash relative to standard cash transfers, which can be conditional or unconditional depending on program design. While not necessarily targeted for emergencies, DC transfers can be particularly valuable during peak-demand times of food insecurity, such as seasonal hunger gaps or crises, by providing households with increased financial flexibility to meet short-term food and nutritional needs.[2,5] None of the studies reviewed specifically assessed resilience indicators, such as the Reduced Coping Strategies Index (rCSI) or household debt levels, in relation to DC transfers.

Cash + Complimentary Services

Cash + Complimentary services also known as "Cash Plus" models combine cash transfers which can be conditional or unconditional with additional support services such as nutrition education, social and behavior change communication, healthcare, or agricultural assistance. Unlike CCTs, which require recipients to meet specific conditions to receive cash, Cash Plus programs provide integrated support to address barriers beyond financial constraints. By addressing multiple determinants of nutrition, these multifaceted initiatives frequently have the greatest impact on nutrition outcomes, particularly in areas where knowledge about dietary choices is limited or access to nutritious food is restricted.[4]

Educational Components of Cash-Based Interventions

Education interventions and sensitization campaigns play a central role in complementing the impact of CBIs on the nutrition of children. Behavior Change Communication (BCC) training and community sensitization are the most common approaches, both aimed at improving caregiver choice-making and education regarding nutrition, hygiene, and child growth.[2] The education component is usually bundled with CBI interventions to confirm healthy health habits and optimize the impact of the cash transfer.

BCC Sessions are usually conducted in conjunction with cash transfers or other food interventions to provide caregivers with functional nutrition and health education, covering topics such as optimal breastfeeding, complementary feeding practices, hand washing, and bed net use. Cooking demonstrations are sometimes conducted on-site to enable caregivers to prepare healthy meals from resources available to them.[1]

As a broader form of Behavior Change Communication (BCC), community sensitization supports CBIs by raising awareness among beneficiaries about the importance of allocating cash toward child nutrition and development. These sessions fill the gap between cash provision and behavior change towards better nutrition outcomes.[4]

A good example of an intervention that integrated BCC and community sensitization was the WINS Program in Sindh Province, Pakistan, in a humanitarian setting. All study participants received monthly group education by research mobilizers.[1,3] Education covered important topics such as:

- Causes of undernutrition
- Benefits of exclusive breastfeeding
- Improved complementary feeding practices
- Food and water hygiene
- Handwashing and sanitation

In addition to micronutrient supplementation and outpatient treatment of severely malnourished children, the WINS program also aimed to educate caregivers with information required to maintain child nutrition and health.[1] With the integration of BCC and community sensitization into CBIs, these interventions can enhance the long-term impact of cash transfers on child development and health.

Gaps Identified in the Literature on Cash-Based Interventions for Child Nutrition

Though cash plus interventions have improved nutritional outcomes compared to cash alone, few of the studies fail to quantify major determinants that lead to malnutrition. Some studies quantify child nutrition status using anthropometric measures of weight-for-height z-scores (WHZ) and height-for-age z-scores (HAZ), but neither household-level food security nor dietary diversity is quantified.[1,3] None of the studies made use of Food Consumption Scores (FCS) and Household Dietary Diversity Scores (HDDS), constraining our ability to identify how household food consumption habits and food availability are affected by economic interventions. Consistency in the measurement of the anthropometric indicator was also absent between the studies, making comparative analysis challenging. Closing these gaps by introducing household-level food security and dietary

diversity indicators in addition to the standard anthropometric ones would contribute more to greater comprehension of CBIs' child nutrition gains over immediate growth-related ones.

Nearly all studies evaluate the short- to medium-term impacts of cash-based interventions on child nutrition and household outcomes and were typically less than one year in length. Some nutrition markers, such as wasting, may react relatively swiftly, while others, such as stunting, require longer intervention before substantial change is noted. There is little data, though, on long-term sustainability of gains following interventions. Whether decreases in stunting and wasting or in dietary variety are maintained long term after the discontinuation of cash transfers is unknown. Further studies are needed to assess the long-term effects of CBIs on child nutrition.

Emerging Research and Protocols in Cash-Based Interventions

There are many emerging studies being proposed and conducted pertaining to cash-based interventions. Recent studies have also published research protocols to evaluate cash-based interventions (CBIs). [8,9] Although most are still in early stages without final outcomes, their designs highlight essential factors for child and maternal nutrition, such as differences in cash delivery methods, program duration, and integration of additional support services. Each study has a unique approach in investigating the impact and challenges of CBIs:

- Huda (2020)[8]: The study, implemented in rural Bangladesh, focuses on conditional cash transfers connected to maternal healthcare visits, evaluating the impact of certain health behaviors, such as exclusive breastfeeding, maternal nutrient intake, and complementary feeding practices can boost nutritional outcomes. It measures dietary improvements and long-term health indicators like stunting and wasting, exploring how health education might directly influence behavior changes [8]
- Martin-Canavate (2023)[9]: This protocol paper aims to assess the cost-effectiveness and cost-efficiency of three interventions to reduce stunting in children under 2 from low-income households in Cunene and Huila, Angola. The interventions include: (1) standard of care (SoC) alone, (2) SoC plus nutritional supplements, and (3) SoC plus cash transfers. The economic evaluation will take a societal perspective to estimate implementation costs for each intervention and costs for participants and families. It will calculate cost-efficiency per woman, child, and household reached and estimate cost-effectiveness by determining the cost per case of stunting averted compared to standard care.

References for Text S6

1 Fenn B, Colbourn T, Dolan C, Pietzsch S, Sangrasi M, Shoham J. Impact evaluation of different cash-based intervention modalities on child and maternal nutritional status in Sindh Province, Pakistan, at 6 mo and at 1 y: A cluster randomised controlled trial. PLoS Med. 2017;14:e1002305. Medline:28542506

2 Trenouth L, Sibson VL, Grijalva-Eternod CS, Golden K, Puett C. Cost and cost-efficiency of unconditional cash transfers in Tahoua, Niger. J Dev Effect. 2023;15:111–23.

3 Trenouth L, Colbourn T, Fenn B, Pietzsch S, Myatt M, Puett C. The cost of preventing undernutrition: cost, cost-efficiency and cost-effectiveness of three cash-based interventions on nutrition outcomes in Dadu, Pakistan. Health Policy Plan. 2018;33:743–54.

4 Puett C, Salpéteur C, Houngbe F, Martínez K, N'Diaye DS, Tonguet-Papucci A. Costs and cost-efficiency of a mobile cash transfer to prevent child undernutrition during the lean season in Burkina Faso: a mixed methods analysis from the MAM'Out randomized controlled trial. Cost Effectiveness and Resource Allocation. 2018;16:13.

5 Doocy S, Tappis H, Lyles E. Are cash-based interventions a feasible approach for expanding humanitarian assistance in Syria? Journal of International Humanitarian Action. 2016;1:13.

6 Norwegian Refugee Council. Cash Alliance Somalia: Final Report. 2023. Available: https://www.nrc.no/globalassets/pdf/reports/food-security-somalia/nrc_cash-alliance_final-report_v6_clean.pdf.

7 Ali MK, Flacking R, Sulaiman M, Osman F. Effects of Nutrition Counselling and Unconditional Cash Transfer on Child Growth and Family Food Security in Internally Displaced Person Camps in Somalia—A Quasi-Experimental Study. Int J Environ Res Public Health. 2022;19:13441.

8 Huda TM, Alam A, Tahsina T, Hasan MM, Iqbal A, Khan J, et al. Shonjibon cash and counselling: a community-based cluster randomised controlled trial to measure the effectiveness of unconditional cash transfers and mobile behaviour change communications to reduce child undernutrition in rural Bangladesh. BMC Public Health. 2020;20:1776.

9 Martin-Cañavate R, Custodio E, Trigo E, Romay-Barja M, Herrador Z, Aguado I, et al. Preventing chronic malnutrition in children under 2 years in rural Angola (MuCCUA trial): protocol for the economic evaluation of a three-arm community cluster randomised controlled trial. BMJ Open. 2023;13:e073349.

Table S17. Strengths and Limitations of Cost Analysis Approach

Strengths	In-depth understanding: The KIIs and focus group discussions allowed for in-depth exploration of the newly introduced concept of societal opportunity costs, which is often difficult to replicate capture through independent quantitative surveys. Furthermore, programme staff often possess nuanced understanding of the context and can provide rich qualitative data on how the intervention affects beneficiaries' choices and behaviours.<br>Contextualized information: The discussions also provided valuable contextual information about the specific circumstances in Hiran and Bay, such as economic realities, and social dynamics, which influence opportunity costs.<br>Access to insider perspectives: SCI staff have direct experience of the intervention and its impact on beneficiaries, and they were able to offer insights into the unintended consequences and hidden costs associated with participation in the programme, where relevant, which may not have been readily apparent.<br>Flexibility and adaptability: The KIIs and focus groups also allowed for flexibility in probing emerging themes and exploring unexpected responses, leading to a richer understanding of the factors affecting opportunity costs and the wider economic evaluation.<br>Triangulation: Information gathered from the discussions were then used to triangulate and validate data collected through other methods (e.g. direct economic costings), thereby increasing the reliability of the wider cost-efficiency findings.
Limitations	Subjectivity and bias: As with any qualitative method, participants (in this case, SCI staff) may have inherent biases due to their involvement in the intervention. As such, they may downplay negative consequences, such as societal impacts, potentially skewing the estimation of opportunity costs.<br>Limited generalizability: Findings from KIIs and the focus groups may not be representative of the wider population in Hiran and Bay. The perspectives of programme staff may not reflect the diverse experiences and viewpoints of all beneficiaries.<br>Difficulty in quantification: While interviews and focus groups can provide valuable qualitative insights, it can be challenging to translate these qualitative narratives into quantifiable measures of opportunity costs. This limitation can hinder precise estimation and comparison of costs across the different R2HC Arms and regions.<br>Time and resource intensive: Conducting in-depth interviews with multiple programme staff is resource-intensive and highly time sensitive to coincide with the convening of the Nairobi event, as such, there was limited sample size of participants, potentially affecting the generalizability of findings.

	Availability of secondary data: Utilizing existing secondary data sources was required for estimating the economic cost of beneficiaries' time, which could have been used for other activities (such as paid work). Sources like labour market surveys, economic reports from governmental or non-governmental organizations, and academic studies to provide data on a realistic hourly wage for the rural Somalia informal market were extremely limited. As such, a combination of the 'Somalia average income per capita (annual)' from a World Data source[1] and Clockify's 'average number of working hours per year (Somalia)'[2] was used to estimate an hourly cost. Using this highly approximate method to quantify the opportunity costs of time spent participating in the intervention will have had a limiting factor on the accuracy and reliability of the societal cost estimations.
Mitigation Approach	Participant selection: Due to the convening of the Nairobi event, the SCI programme informants available were ideally placed to have diverse roles and experiences within the programme to capture a range of perspectives.<br>Use of structured guides: To develop a standardized approach to measuring societal costs, guides were developed with clear and specific questions to ensure consistency and reduce bias (attached in email).<br>Triangulation of data sources: The combination of the KIIs and focus group discussions, with secondary data from programme documentation to validate findings and enhance the reliability of estimations. For example, the SCI programme staff were able to confirm the hourly wage estimate (sourced from secondary data) was realistic for the intervention context.<br>Quality facilitation: All KIIs and focus groups were facilitated by the lead economic evaluator.

Box 1. Trial Inclusion and Exclusion Criteria

Study Participant	Inclusion Criteria	Exclusion Criteria
Mothers of children 6 to 59 months	Enrolled in Save the Children Cash Plus for Nutrition program<br>Have a child that is 6–59 months of age at baseline<br>Subject who is voluntarily willing to participate by signing the consent form	Currently receiving treatment for MAM* or SAM**<br>Had an episode of severe wasting (SAM) in the past 12 months
Children 6–59 months	Enrolled in Save the Children Cash Plus for Nutrition program<br>6–59 months of age at baseline<br>Mother is enrolled in the study	Receiving treatment for MAM or SAM at baseline<br>Had an episode of severe wasting (SAM) in the past 12 months

*Moderate acute malnutrition. **Severe acute malnutrition.
